# Porous Silicon Nanocarriers with Stimulus‐Cleavable Linkers for Effective Cancer Therapy

**DOI:** 10.1002/adhm.202200076

**Published:** 2022-04-03

**Authors:** Yufei Xue, Hua Bai, Bo Peng, Terence Tieu, Jiamin Jiang, Shiping Hao, Panpan Li, Mark Richardson, Jonathan Baell, Helmut Thissen, Anna Cifuentes, Lin Li, Nicolas H. Voelcker

**Affiliations:** ^1^ Frontiers Science Center for Flexible Electrons Xi'an institute of Flexible Electrons (IFE) and Xi'an institute of Biomedical Materials and Engineering Northwestern Polytechnical University (NPU) 127 West Youyi Road Xi'an 710072 China; ^2^ Drug Delivery, Disposition and Dynamics Monash institute of Pharmaceutical Sciences Monash University Parkville Victoria 3052 Australia; ^3^ Commonwealth Scientific and Industrial Research Organisation (CSIRO) Clayton Victoria 3168 Australia; ^4^ Melbourne Centre for Nanofabrication Victorian Node of the Australian National Fabrication Facility Clayton Victoria 3168 Australia; ^5^ Department of Materials Science and Engineering Monash University Clayton Victoria 3168 Australia

**Keywords:** anticancer, controlled drug release, porous silicon nanoparticles (pSiNPs), stimulus‐cleavable linker, surface chemistry

## Abstract

Porous silicon nanoparticles (pSiNPs) are widely utilized as drug carriers due to their excellent biocompatibility, large surface area, and versatile surface chemistry. However, the dispersion in pore size and biodegradability of pSiNPs arguably have hindered the application of pSiNPs for controlled drug release. Here, a step‐changing solution to this problem is described involving the design, synthesis, and application of three different linker‐drug conjugates comprising anticancer drug doxorubicin (DOX) and different stimulus‐cleavable linkers (SCLs) including the photocleavable linker (*ortho*‐nitrobenzyl), pH‐cleavable linker (hydrazone), and enzyme‐cleavable linker (*β*‐glucuronide). These SCL‐DOX conjugates are covalently attached to the surface of pSiNP via copper (I)‐catalyzed alkyne‐azide cycloaddition (CuAAC, i.e., click reaction) to afford pSiNP‐SCL‐DOXs. The mass loading of the covalent conjugation approach for pSiNP‐SCL‐DOX reaches over 250 µg of DOX per mg of pSiNPs, which is notably twice the mass loading achieved by noncovalent loading. Moreover, the covalent conjugation between SCL‐DOX and pSiNPs endows the pSiNPs with excellent stability and highly controlled release behavior. When tested in both in vitro and in vivo tumor models, the pSiNP‐SCL‐DOXs induces excellent tumor growth inhibition.

## Graphical abstract

A covalent binding strategy is demonstrated to conjugate anticancer drug to the surface of porous silicon nanoparticles through stimulus‐cleavable linkers and this unique technology realizes high drug content and stimulus‐controlled drug release from porous silicon nanoparticles.

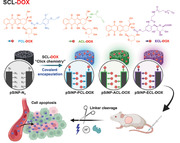

## 1 Introduction

The medical application of nanotechnology known as nanomedicine affords the possibility of delivering small molecule drugs to specific cells within the body through the use of nanoparticles (NPs).^[^ ^1^ ^]^ Various NP‐based drug delivery systems (DDSs) with different compositions have been investigated, such as polymeric NPs,^[^ ^2^ ^]^ lipid‐based NPs,^[^ ^3^ ^]^ dendrimers,^[^ ^4^ ^]^ gold NPs,^[^ ^5^ ^]^ mesoporous silica NPs (MSNs),^[^ ^6^ ^]^ and porous silicon NPs (pSiNPs).^[^ ^7^ ^]^ Among these DDSs, both MSNs and pSiNPs are silicon‐based and offer advantages to the biomedical field owing to their large surface area, high porosity, versatile surface chemistry, unique optical, and physical properties.^[^ ^8^ ^]^ However, often mistaken for its counterpart MSNs which are synthesized from organosilicons, pSiNPs are fabricated by anodizing silicon wafers in hydrofluoric acid (HF) solution followed by fracturing the resulting porous films. Compared to the very slow degradation of MSNs, pSiNPs are readily degraded by oxidative hydrolysis into nontoxic orthosilicic acid,^[^ ^9^ ^]^ making pSiNPs a particularly exciting bionanomaterial for drug delivery applications.

Owing to the abovementioned advantages, pSiNPs have been applied in numerous therapeutic delivery applications, using small molecules, nucleic acids, and proteins alone or in combination.^[^ ^10^ ^]^ Importantly, nanomedicine that can deliver small molecule drugs in a spatiotemporally controlled manner enhances the therapeutic efficacy of the drugs and reduces their systemic toxicity.^[^ ^11^ ^]^ Despite the range of successes previously reported related to controlled release, stimulus‐responsive drug release from pSiNPs has not been widely investigated.^[^ ^7^, ^12^ ^]^ As a result of the distribution in pore size and high biodegradability, the molecular gate concept that is widely used in stimulus‐responsive controlled release strategies involving MSNs has been rarely applied to pSiNPs.^[^ ^13^ ^]^ The loading of therapeutics in pSiNPs has predominantly been dependent on noncovalent interactions within the porous structure such as through electrostatic interaction,^[^ ^14^ ^]^ *π*‐*π* interaction,^[^ ^15^ ^]^ and oxidation‐chelate interaction^[^ ^16^ ^]^ between the therapeutic agent and the surface. Our group also reported the coating of the surface of pSiNPs with stimulus‐responsive polymers to achieve the controlled drug release.^[^ ^17^ ^]^ However, the aforementioned methods still suffered from the uncontrolled release or leakage of the cargo, which in turn results in higher toxicity for healthy tissues and organs, significantly impacting therapeutic efficiency, dosage and controlled release ability.^[^ ^1^, ^18^ ^]^

We recently summarized the applications of stimulus‐responsive linkers (SCLs) in achieving controlled drug release.^[^ ^19^ ^]^ SCLs are chemical structures with sensitivity to single/multiple stimuli. Cleavage of the linker structure only occurs after exposure to the corresponding stimulus. In order to achieve controlled drug release behavior, the incorporation of SCLs has been applied to conjugate small molecule drugs to a range of drug carriers and has afforded exquisite control over the release profile in response to exposure to the relevant stimulus.^[^ ^20^ ^]^ It is noteworthy that conjugation of SCLs to drug molecule requires the latter to feature specific functional groups (e.g., alcohol, amine, carboxylic acid, sulfide, etc.).^[^ ^19^ ^]^

**Scheme 1:**
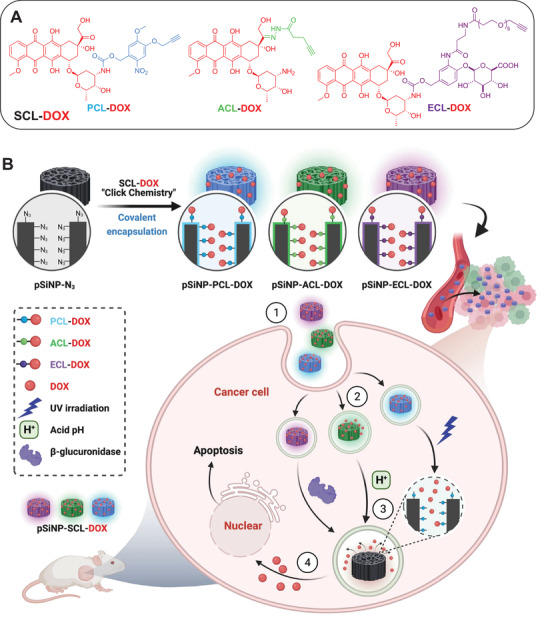
A) Chemical structures of three SCL‐DOXs: photocleavable linker‐DOX conjugate (**PCL‐DOX**); enzyme (*β*‐glucuronidase)‐cleavable linker‐DOX conjugate (**ECL‐DOX**) and acid‐cleavable linker‐DOX conjugate (**ACL‐DOX**). B) Covalent encapsulation was realized by click reaction between different SCL‐DOXs and the azide‐functionalized pSiNPs (**pSiNP‐N_3_**) to generate **pSiNP‐PCL‐DOX** (blue), **pSiNP‐ACL‐DOX** (green), and **pSiNP‐ECL‐DOX** (purple), respectively. The pSiNPs were taken up by the cells (steps 1 and 2) via endocytosis. The linkers were then cleaved via different mechanism (enzyme exposure, acidic pH, and photoirradiation, step 3), resulting in the release of DOX and inducing apoptosis in the exposed cells (step 4). Created with BioRender.com.

Herein, we introduce an approach where a small molecule drug was covalently attached to the drug carrier pSiNPs via three different types of SCL (**Scheme** **1**). Loading drugs via covalent conjugation enables significantly higher and more stable drug loading and controlled release of drugs. The anticancer drug doxorubicin (DOX) was conjugated to three different SCLs that can be cleaved by either photoirradiation **(PCL‐DOX)**, acidic pH (**ACL‐DOX**), or the enzyme *β*‐glucuronidase (**ECL‐DOX**). The linker‐drug conjugates (SCL‐DOXs) were further covalently attached to the surface of pSiNPs via high yielding click reaction. The conjugation of the SCL‐DOXs with pSiNPs resulted in high drug mass loadings (up to 250.40 ± 21.90 µg of DOX per mg of pSiNPs, 23.7% of the theoretical maximum loading capacity, LC_max_), stable drug loading (leakage was limited to 5.6% of the drug payload within the 48 h in phosphate buffered saline (PBS) at 37 °C), and excellent controlled release profiles. Although a similar approach has been used in MSNs and achieved a certain degree of controlled release behavior, the reported mass loadings (from 11.02 to 32.67 µg of DOX per mg of MSNs) is far inferior to that obtained in this study.^[^ ^21^ ^]^ The pSiNP‐SCL‐DOXs exhibited stimulus‐dependent anticancer activity both in vitro and in vivo. Notably, photo‐ and pH‐sensitive nanoconjugates (**pSiNP‐PCL‐DOX** and **pSiNP‐ACL‐DOX**, respectively) had higher in vivo tumor growth inhibitory activities compared to the free DOX administrated at the same cargo concentration. Therefore, we have demonstrated that covalent conjugation strategies can afford significantly improved controlled drug release profiles, which has been a consistent challenge for pSiNP‐based drug delivery.

## 2 Results and Discussion

The large surface area of pSiNPs (up to 600 m^2^ g^−1^) allows the covalent attachment of a large amount of drugs and photosensitizers to the surface of pSiNPs, which has resulted in excellent therapeutic performance when using pSiNPs as drug carrier.^[^ ^22^ ^]^ We hypothesized that covalent conjugation of drugs via SCLs may result in a similar mass loading to the traditional drug loading method but also afford stimulus‐responsive controlled release, regardless of the biodegradability of the drug carrier. We first used mathematical models to estimate the loading capacity (LC) of covalent conjugation approach (Supporting Information). The result showed that by immobilizing drugs on both the internal and external surface of pSiNPs (18.6 nm average pore size), the covalent drug conjugation can achieve a theoretical loading capacity (LC_surface_) of 566.04 µg of DOX per mg of pSiNPs, which is assuming densely packed monolayers of drug on the pore and external surface. Although the theoretical maximum loading capacity (LC_max_) achieved by noncovalent drug loading approach is much higher at 1056.6 µg of DOX per mg of pSiNPs (assuming drug fills the volume of the pores of pSiNPs rather than just being adsorbed to the pore surface), the reported values for loading DOX into pSiNPs are much lower (from 43.80 to 126.13 µg of DOX per mg of pSiNPs).^[^ ^23^ ^]^ Therefore, we anticipated that our covalent conjugation method could reach or exceed the drug mass loadings achieved by the noncovalent encapsulation method.

**Figure 1:**
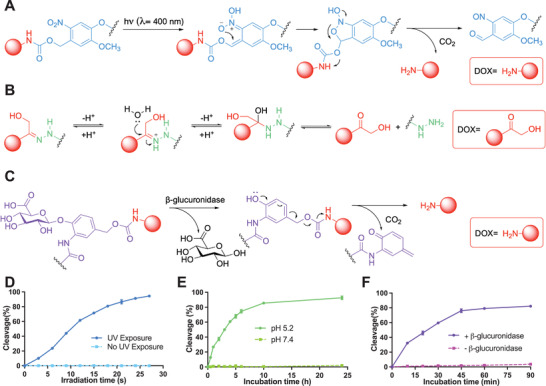
Stimulus‐cleavage of SCL‐DOXs. Mechanism of the cleavage of A) **PCL‐DOX**, B) **ACL‐DOX**, and C) **ECL‐DOX**. Linker cleavage and DOX release kinetics of D) **PCL‐DOX**, E) **ACL‐DOX**, and F) **ECL‐DOX** with and without presence of the corresponding stimuli. Data are shown as mean ± SD (*N* = 3).

For the choice of SCLs, we selected three SCLs as a proof‐of‐concept, which are sensitive to different stimuli covering both exogeneous and endogenous stimuli (**Figure** **1**). For the exogeneous stimulus, we chose to use light as an external trigger due to its noninvasiveness, ease of tuning, and on‐demand nature.^[^ ^24^ ^]^ The most widely used *ortho*‐nitrobenzyl (ONB) group was chosen as the core structure of the photocleavable linker (PCL).^[^ ^25^ ^]^ Whilst UV light‐cleavable linkers are not ideal for in vivo use, several UV‐responsive DDSs have been reported to release the cargo upon photoirradiation in in vivo tests at the wavelength of 400 nm.^[^ ^26^ ^]^ Apart from using an external stimulus, endogenous stimuli were also chosen due to the elevated level of biomolecules or abnormal microenvironments that are widely observed in many tumor tissues. In this regard, as a physiological parameter that varies across different organelles, pH is a promising endogenous stimulus for targeted drug release. Here we designed a hydrazone‐based acid cleavable linker (ACL) owing to its high susceptibility to acidic conditions (such as in endosomes and lysosomes) and its stability in a neutral pH environment.^[^ ^21d^ ^]^ In addition, an enzyme‐cleavable linker (ECL) which is sensitive to *β*‐glucuronidase, a lysosomal enzyme overexpressed in many types of cancers, was chosen.^[^ ^27^ ^]^

We first synthesized three SCL‐DOXs according to methods adapted from the literature^[^ ^28^ ^]^ and the synthesis route is illustrated in Schemes S1–S3 in the Supporting Information. The chemical structures of **PCL‐DOX**, **ACL‐DOX**, and **ECL‐DOX** were fully characterized through ^1^H NMR, ^13^C NMR, and high‐resolution mass spectrometry (Supporting Information). We then confirmed the stimulus‐responsiveness of each SCL‐DOX by high‐performance liquid chromatography (HPLC) (Figure S1, Supporting Information, release mechanism shown in Figure 1A–C). **PCL‐DOX** remained stable in PBS in the dark. The photoirradiation (*λ* = 400 nm, 849 mW cm^−2^) induced cleavage of the **PCL‐DOX** in a dose‐dependent manner. As shown in Figure 1D, 94.4% ± 1.0% of the **PCL‐DOX** composite was cleaved over the 24 s photoirradiation (133 J), demonstrating the susceptibility of ONB linkage to UV light. The sensitivity of **ACL‐DOX** to an acidic environment mimicking endosomes and lysosomes was evaluated in sodium acetate buffer (0.1 m, pH 5.2) at 37 °C. As shown in Figure 1E, the cleavage of **ACL‐DOX** reached 85.5% ± 1.0% within 10 h, while it remained stable at neutral pH in PBS (0.1 m, pH 7.4). We finally examined the sensitivity of **ECL‐DOX** to *β*‐glucuronidase (250 U mL^−1^) at different time points in PBS at 37 °C. Approximately 76.0% ± 1.5% cleavage of **ECL‐DOX** was observed within 45 min incubation with *β*‐glucuronidase (Figure 1F), suggesting its high susceptibility to *β*‐glucuronidase. In contrast, no cleavage of **ECL‐DOX** was detected in the absence of *β*‐glucuronidase.

Once we confirmed the robust responsiveness of the SCL‐DOXs to stimuli, we next covalently attached them to pSiNPs. pSiNPs were fabricated by anodic electrochemical etching as previously reported.^[^ ^10b^ ^]^ Following electrochemical etching, the pSi film was fractured into pSiNPs by ultrasonication. Subsequently, size selection via ultracentrifugation allowed for the selection of pSiNPs with a size of ≈200 nm. N_2_ adsorption‐desorption isotherms of pSiNPs showed an average pore size of 18.6 nm, total surface area of 489.17 m^2^ g^−1^ and pore volume of 0.741 cm^3^ g^−1^.^[^ ^10^, ^29^ ^]^ Freshly anodized pSi remains highly reactive and unstable due to the reactive hydrogen terminated surface (Si‐H). To prevent rapid degradation and present suitable surface functional groups, pSiNPs were reacted with 11‐bromo‐1‐undecene via thermal hydrosilylation to form **pSiNP‐Br** (Scheme S4, Supporting Information). Afterward, the bromine groups on **pSiNP‐Br** were replaced with azide groups through the reaction with sodium azide to afford **pSiNP‐N_3_**, which were finally conjugated with each of the SCL‐DOXs via click reaction to form **pSiNP‐PCL‐DOX**, **pSiNP‐ACL‐DOX** and **pSiNP‐ECL‐DOX**, respectively (Scheme S4, Supporting Information).

**Figure 2:**
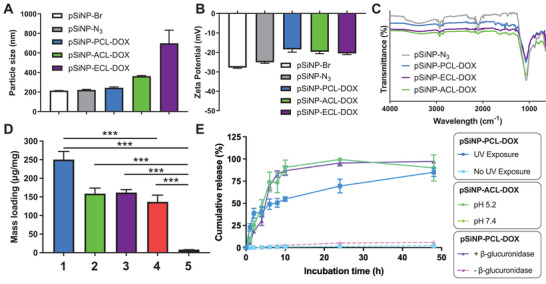
A) Particle size measured by DLS and B) *ζ*‐potential of **pSiNP‐Br**, **pSiNP‐N_3_**, **pSiNP‐PCL‐DOX**, **pSiNP‐ACL‐DOX**, and **pSiNP‐ECL‐DOX** in deionized H_2_O. Data shown as mean ± SD (*N* = 3). C) FTIR spectra of **pSiNP‐N_3_**, **pSiNP‐PCL‐DOX**, **pSiNP‐ACL‐DOX**, and **pSiNP‐ECL‐DOX**. D) Loading capacity summary of **pSiNP‐PCL‐DOX**, **pSiNP‐ACL‐DOX**, **pSiNP‐ECL‐DOX**, and **DOX@pSiNP‐UA** (PBS/DMSO wash). The loading capacity was determined by TGA and/or fluorescence spectroscopy. 1: **pSiNP‐PCL‐DOX**; 2. **pSiNP‐ACL‐DOX**; 3. **pSiNP‐ECL‐DOX**; 4. **DOX@pSiNP‐UA** (PBS wash); 5. **DOX@pSiNP‐UA** (DMSO wash). Data shown as mean ± SD (*N* = 3). Student's *t*‐test, P***<0.001. Release kinetic of DOX from E) cumulative release of pSiNP‐SCL‐DOX upon the treatment of corresponding stimuli. Data shown as mean ± SD (*N* = 3).

The morphologies and sizes of the resulting pSiNP‐SCL‐DOXs were characterized by transmission electron microscopy (TEM) and dynamic light scattering (DLS). The average particle size of both **pSiNP‐N_3_** and **pSiNP‐PCL‐DOX** was confirmed to be ≈200 nm as shown in TEM images (Figure S2A,B, Supporting Information) and DLS measurements (**Figure** **2**A). Meanwhile, the particle sizes of **pSiNP‐ACL‐DOX** (370 nm) and **pSiNP‐ECL‐DOX** (700 nm) which were measured by DLS exhibited significantly larger sizes. This may be attributed to the hydrophobicity of the **ACL‐DOX** and **ECL‐DOX** on the surface which is expected to lead to a small extent of clustering of the particles in water. Interestingly, the TEM of pSiNP‐SCL‐DOXs exhibited a layer of light grey haze both in the internal and external surface of pSiNPs which may reflect the presence of the SCL‐DOXs (Figure S2A–D, Supporting Information). Further DLS experiments were performed to evaluate the colloidal stability of **pSiNP‐N_3_** and pSiNP‐SCL‐DOXs in PBS, Dulbecco's modified eagle medium (DMEM) with fetal bovine serum (FBS) and DMEM with bovine serum albumin (BSA). As summarized in Figure S3 in the Supporting Information, the **pSiNP‐PCL‐DOX** remained the good dispersion state in all three biological solutions, while the **pSiNP‐N_3_** and **pSiNP‐ACL‐DOX** underwent aggregation to some extent in these solutions.

To further corroborate the successful functionalization of the surface of pSiNPs, *ζ*‐potential and Fourier transform infrared spectroscopy (FTIR) measurements were acquired. The *ζ* potential data show that **pSiNP‐N_3_** is negative (−25.3 ± 0.4 mV), and that negative charge was decreased after conjugating with SCL‐DOXs, to around −20 mV in all three formulations (Figure 2B). The successful SCL‐DOX conjugation on pSiNPs was further confirmed by the FTIR analysis (Figure 2C). The C—H stretches at 2940 and 2980 cm^–1^ appeared on all the surface‐functionalized samples were ascribed to the hydrocarbon chains introduced on the surface after thermal hydrosilylation. The **pSiNP‐N_3_** contained a N=N=N stretch mode at 2100 cm^–1^, which is further shown to be absent in the samples after click reaction.

The large surface area and high mass loading of the pSiNPs is one of the key advantages of pSiNPs as a drug carrier. To confirm that the loading efficiency of DOX via covalent attachment with SCL is comparable to traditional loading strategies, we first measured the mass loading of pSiNP‐SCL‐DOXs using fluorescence spectroscopy. pSiNP‐SCL‐DOXs were subjected to the corresponding stimulus, which was followed by thorough sonication to initiate the total release of DOX contents from the pSiNPs. The amount of released DOX in the supernatant was collected and verified by fluorescence spectroscopy (DOX, *λ*_ex/em_ = 470/595 nm) to provide the corresponding mass loading of each pSiNP‐SCL‐DOX (Figure S4, Supporting Information). The mass loading of **pSiNP‐PCL‐ DOX** was verified to be 250.40 ± 21.90 µg of DOX per mg of pSiNPs, while that of **pSiNP‐ACL‐DOX** and **pSiNP‐ECL‐DOX** were slightly lower at 158.95 ± 14.75 and 161.50 ± 8.10 µg of DOX per mg of pSiNPs, respectively. Thermogravimetric analysis (TGA) was simultaneously carried out to further confirm the mass loading of pSiNP‐SCL‐DOXs, which showed consistent results with those obtained by fluorescence spectroscopy (Figure S5A,B, Supporting Information). Notably, the mass loading of **pSiNP‐PCL‐DOX** reaches 44.2% of the LC_surface_ and 23.7% of the LC_max_. In order to compare with the noncovalent loading method, we used undecylenic acid‐functionalized pSiNPs (**pSiNP‐UA**) as an example of physical absorption which is able to encapsulate DOX via the interaction between the carboxylic acid and the primary amine from DOX.^[^ ^30^ ^]^ After the incubation of **pSiNP‐UA** with DOX overnight (**DOX@pSiNP‐UA**) and the gentle wash with PBS, a mass loading of 136.70 ± 18.10 µg of DOX per mg of pSiNPs was determined, which is 12.9% of the LC_max_ (Figure S5C, Supporting Information). In summary, the mass loading of **pSiNP‐PCL‐DOX** is ≈7–22 times higher than that of the similar approaches based on MSN (from 11.02 to 32.67 µg of DOX per mg of MSNs)^[^ ^21^ ^]^ and approximately twice as much as the noncovalent loading DOX in pSiNPs (**DOX@pSiNP‐UA**) and other reported ones.^[^ ^14^, ^23^ ^]^ The difference in the mass loadings of pSiNP‐SCL‐DOXs might explained by the different sterics and hydrophobicities of the SCL‐DOXs. Additionally, by applying the same washing protocol as pSiNP‐SCL‐DOX where the **pSiNP‐UA** were thoroughly washed via sonication with dimethyl sulfoxide (DMSO), the drug mass loading was 10.10 µg of DOX per mg of pSiNPs, which is 1.0% of LC_max_ (Figure 2D and Figure S5C, Supporting Information). These results demonstrate the superior mass loading and stability of our covalent conjugation approach compared to an entrapment strategy.

Following the demonstration of the stimulus‐responsiveness of SCL‐DOXs and high drug loading efficiency of covalent conjugation, the controlled release profiles of the pSiNP‐SCL‐DOXs were evaluated. Each pSiNP‐SCL‐DOX was treated with the corresponding stimulus and the supernatant contents of the aliquot at each time point was analyzed by fluorescence spectroscopy (*λ*_ex/em_ = 470/595 nm). The release of DOX from **pSiNP‐PCL‐DOX** was photodependent. Upon the exposure of photoirradiation (*λ* = 400 nm, 133 J corresponding to 24 s of exposure), more than 80% of DOX was released from **pSiNP‐PCL‐DOX** over 48 h. Although the cleavage of the linker occurred within seconds, the release kinetics of DOX were similar to DOX noncovalently loaded into NPs.^[^ ^14^, ^23^ ^]^ In contrast, only 2.2% of DOX was released over the same period of incubation time in the dark (Figure 2E). For **pSiNP‐ACL‐DOX**, under the acidic environment (pH 5.2), the amount of DOX released from **pSiNP‐ACL‐DOX** was 82% after 48 h, while the cumulative release of DOX after 48 h was only 2.2% at physiological pH (7.4) (Figure 2E). Lastly, the controlled release profile of **pSiNP‐ECL‐DOX** was examined by the addition of *β*‐glucuronidase (250 U mL^−1^) and 65.1% of DOX was released within the first 6 h. A subsequent 33% of DOX was released over the next 42 h (98.1% release in total). In contrast, only 5.6% of loaded DOX was released after 48 h incubation in the absence of *β*‐glucuronidase (Figure 2E). The small amount of drug release in the control groups for all three pSiNP‐SCL‐DOXs was attributed to the degradation of pSiNPs.^[^ ^31^ ^]^ As summarized in Figure S5D in the Supporting Information, the release of DOX from pSiNP‐SCL‐DOXs is stimulus dependent and further demonstrates the excellent properties of the covalent conjugation method.

Inspired by the outstanding controlled release profile of pSiNP‐SCL‐DOXs, we decided to further evaluate the controlled intracellular delivery of DOX on human cervical cancer (HeLa cell line) cells. For the in vitro controlled release, UV‐irradiated **pSiNP‐PCL‐DOX** was observed to be internalized by HeLa cells after 1 h of incubation (Figure S6A, Supporting Information) and strong signal of DOX was detected in the cytoplasm at 12 h. **pSiNP‐ACL‐DOX** were also rapidly taken up by the HeLa cells, as evidenced by the appearance of the fluorescence signal from DOX in the cytoplasm after only 1 h of incubation (Figure S6B, Supporting Information). DOX fluorescence was observed in the cytoplasm of HeLa cells treated with **pSiNP‐ECL‐DOX** and *β*‐glucuronidase (250 U mL^−1^) after 1 h of incubation and much stronger DOX signal was observed in the cytoplasm as incubation time extended to 12 h, suggesting that **pSiNP‐ECL‐DOX** has been uptaken and accumulated in the HeLa cells (Figure S6C, Supporting Information). In conclusion, all three pSiNP‐SCL‐DOXs can efficiently deliver DOX into HeLa cells, where the SCLs underwent cleavage in the presence of the corresponding stimulus.

**Figure 3:**
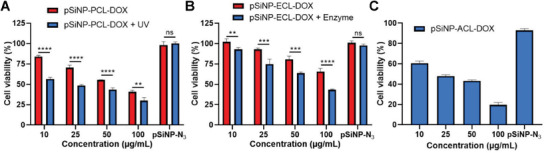
Cytotoxicity of pSiNP‐SCL‐DOXs in HeLa cells. A) Cell viability of HeLa cells treated with different concentrations of **pSiNP‐PCL‐DOX** for 48 h, with (red) or without (blue) photoirradiation. B) Cell viability of HeLa cells treated with different concentrations of **pSiNP‐ECL‐DOX** for 48 h, with (red) or without (blue) *β*‐glucuronidase incubation at physiological pH. C) Cell viability of HeLa cells treated with different concentrations of **pSiNP‐ACL‐DOX** for 48 h at physiological pH. Data shown as mean ± SD (*N* = 4). Student's *t*‐test, ns: not significant, P**<0.01, P***<0.001, P****<0.0001.

Having confirmed the controlled release and cellular uptake of the pSiNP‐SCL‐DOXs, we next evaluated the cytotoxicity of all three pSiNP‐SCL‐DOXs via a luminescence‐based cell viability assay. We investigated the effect of all three pSiNP‐SCL‐DOXs on both HeLa and human melanoma (C32 cell line) cells after 48 h of incubation. As shown in **Figure** **3**A and Figure S7A in the Supporting Information, cell viability results showed that even without photoirradiation, **pSiNP‐PCL‐DOX** still induced moderate cytotoxicity as the concentration of pSiNPs increased. This was to be expected as the benzyl nitro group on PCL has also been reported to be responsive to hypoxia.^[^ ^32^ ^]^ Upon exposure to photoirradiation, the cytotoxicity of **pSiNP‐PCL‐DOX** increased significantly at all concentrations to both cell lines, killing more than 70% cells at the concentration of 100 µg mL^−1^. Importantly, when the cells that had received the same dosage of photoirradiation were incubated with **pSiNP‐N_3_** at the concentration of 50 µg mL^−1^, no cytotoxicity was observed (Figure 3A and Figure S7A, Supporting Information). This confirmed that neither the photoirradiation nor pSiNPs caused any cytotoxicity, thus the cytotoxicity from **pSiNP‐PCL‐DOX** was due to the controlled release of DOX. Similarly, at low concentrations, **pSiNP‐ECL‐DOX** showed no obvious cytotoxicity to both cell lines after incubation for 48 h (Figure 3B and Figure S7B, Supporting Information). As extracellular *β*‐glucuronidase is a biomarker of a wide range of cancers, to simulate the tumor microenvironment, exogenous *β*‐glucuronidase was added to the cell culture.^[^ ^33^ ^]^ As expected, addition of *β*‐glucuronidase at the concentration of 250 U mL^−1^ restored the antiproliferative effect of **pSiNP‐ECL‐DOX** to both cell lines (Figure 3B and Figure S7B, Supporting Information). On the other hand, the addition of exogenous *β*‐glucuronidase with **pSiNP‐N_3_** did not induce any changes in cell viability. Lastly, we evaluated the cytotoxicity of **pSiNP‐ACL‐DOX** against HeLa and C32 cells. The pH values of late endosome and subsequent lysosomes in cells range between 4.0 and 5.5^[^ ^34^ ^]^ and fit the required cleavage pH for hydrazone moieties. As expected, **pSiNP‐ACL‐DOX** exhibited high cytotoxicity in a concentration‐dependent manner and reached ≈80% cell killing at concentration of 100 µg mL^−1^ in both cell‐lines (Figure 3C and Figure S7C, Supporting Information).

**Figure 4:**
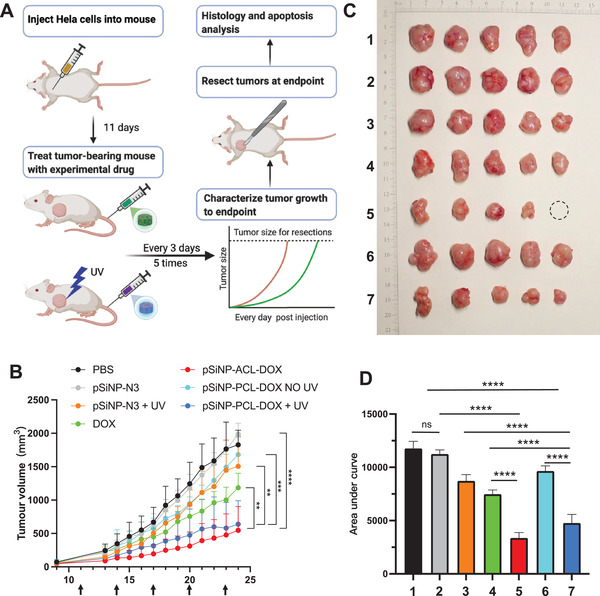
In vivo antitumor efficacy in HeLa‐tumor‐bearing mice. A) Timeframe of the in vivo study. B) Tumor volume changes of HeLa‐tumor‐bearing mice after tail vein injection of PBS, **pSiNP‐N_3_** (no photoirradiation) (4 mg kg^−1^), **pSiNP‐N_3_** (4 mg kg^−1^) + photoirradiation, DOX (1 mg kg^−1^), **pSiNP‐ACL‐DOX** (6.1 mg kg^−1^), **pSiNP‐PCL‐DOX** (no photoirradiation) (7.3 mg kg^−1^), and **pSiNP‐PCL‐DOX** (7.3 mg kg^−1^) + photoirradiation administered on day 11, 14, 17, 20, and 23. **pSiNP‐PCL‐DOX** and **pSiNP‐ACL‐DOX** were administrated to the mice with doses of the equimolar dose equivalents of 1 mg kg^−1^ DOX. Arrow means that the mice were dosed. C) The images of tumors harvested from the mice on day 24 (1: PBS, 2: **pSiNP‐N_3_** (no photoirradiation), 3: **pSiNP‐N_3_** + photoirradiation, 4: DOX, 5: **pSiNP‐ACL‐DOX**, 6: **pSiNP‐PCL‐DOX** (no photoirradiation), 7: **pSiNP‐PCL‐DOX** + photoirradiation). Circle indicates that the tumor was eradicated after the experiment. D) Relative tumor volumes (converted from areas under the curves in Figure 6B) on day 24. Data shown as mean ± SD (*N* = 5). Student's *t*‐test, ns: not significant, P**<0.01, P***<0.001, P****<0.0001. Created with BioRender.com.

Finally, the in vivo antitumor efficiency of pSiNP‐SCL‐DOXs was evaluated using HeLa‐tumor‐bearing BALB/c nude mice. Free DOX, **pSiNP‐PCL‐DOX**, **pSiNP‐ACL‐DOX**, **pSiNP‐N_3_**, and PBS (as a control) were administrated to the mice via intravenous injection with doses of 1 mg kg^−1^ DOX and equimolar dose equivalents of **pSiNP‐PCL‐DOX** and **pSiNP‐ACL‐DOX**, respectively (**Figure** **4**A). Due to the mediocre antitumor effect of **pSiNP‐ECL‐DOX** in HeLa cells, we decided not to include **pSiNP‐ECL‐DOX** in the in vivo experiments. As shown in Figure 4B, free DOX exhibited a moderate tumor growth inhibitory activity compared to control groups (PBS). Notably, **pSiNP‐N_3_** exhibited almost no tumor inhibition effect, further demonstrating that pSiNP is a biocompatible platform for drug delivery. The mice that were administrated with **pSiNP‐PCL‐DOX** but photoirradiation‐free only showed very minor tumor growth inhibition, which correlated well to the in vitro cytotoxicity study. On the other hand, the group that were treated with **pSiNP‐PCL‐DOX** with the exposure of photoirradiation exhibited strong tumor growth inhibition. This is consistent with some other UV‐responsive DDSs at the similar wavelength used here.^[^ ^26^ ^]^ The in vivo antitumor effect of **pSiNP‐PCL‐DOX** upon photoirradiation were significantly stronger than the groups administered with PBS, **pSiNP‐N_3_**, and **pSiNP‐N_3_** with photoirradiation. These results demonstrate that the tumor growth inhibitory activity of **pSiNP‐PCL‐DOX** mainly results from the phototriggered DOX release, rather than the hypoxia and the photocytotoxicity. Similarly, **pSiNP‐ACL‐DOX** showed the strongest antitumor efficacy among all the formulations. More importantly, both **pSiNP‐ACL‐DOX** and **pSiNP‐PCL‐DOX** exhibited significantly stronger tumor growth inhibitory activities than free DOX (Figure 4C,D). We speculated that free DOX was nonselectively distributed throughout the body due to the lack of tumor‐targeting ability, leading to reduced antitumor efficiency.^[^ ^35^ ^]^ In contrast, **pSiNP‐PCL‐DOX** and **pSiNP‐ACL‐DOX** enabled the accumulation of drug at the tumor site due to the enhanced permeability and retention effect, and together with the highly controlled drug release ability, they exhibited a more potent tumor‐inhibiting activity with regard to the control. The slightly lower cytotoxicity of **pSiNP‐PCL‐DOX** as compared with **pSiNP‐ACL‐DOX** could be due to the low tissue penetration depth of UV light, and this will be addressed in our future work via the incorporation linkers reacting to exogenous stimuli with deeper tissue penetration like near‐IR light^[^ ^36^ ^]^ and X‐rays.^[^ ^37^ ^]^

Lastly, histological analysis results further confirmed the significantly higher antitumor effect of pSiNP‐SCL‐DOXs. DOX is known to intercalate within DNA strands, which subsequently inhibits topoisomerase II and induces apoptosis.^[^ ^38^ ^]^ The apoptosis of tumor cells was examined by immunofluorescence staining of terminal deoxynucleotidyl transferase dUTP nick end labeling (TUNEL), shown in Figure S8A in the Supporting Information. The TUNEL assay revealed that the highest cell apoptosis occurs in tumors from the **pSiNP‐ACL‐DOX**‐treated group, compared with PBS and **pSiNP‐N_3_** groups where limited apoptotic cells were found. On the other hand, high levels of apoptosis were observed in the tumors harvested from mice treated with **pSiNP‐PCL‐DOX** with photoirradiation, while tumors in the **pSiNP‐PCL‐DOX** without photoirradiation group exhibited weak green fluorescence, indicating only minor levels of apoptosis. The hematoxylin and eosin (H&E) staining analysis of isolated tumors showed extensive tumor destruction in groups treated with **pSiNP‐ACL‐DOX** and **pSiNP‐PCL‐DOX** with photoirradiation (Figure S8B, Supporting Information). By contrast, the group receiving **pSiNP‐PCL‐DOX** without photoirradiation showed no significant cytotoxicity and was similar to that of PBS, **pSiNP‐N_3_** without and with photoirradiation groups.

## 3 Conclusions

In conclusion, we demonstrated a drug loading strategy by covalently conjugating drugs directly on the surface of nanocarriers via three different types of stimulus‐cleavable linkers (SCLs). By using pSiNPs as the drug carrier, this covalent conjugation approach was demonstrated to feature superior properties in regard to the high mass loading (up to 250.40 ± 21.90 µg of DOX per mg of pSiNPs, 23.7% of the theoretical maximum loading capacity, LC_max_), payload stability and controlled drug release compared to the traditional physical drug loading via electrostatic interactions. The highly controlled release behavior of all three different pSiNP‐SCL‐DOXs was confirmed by the in vitro cell culture experiments. The in vivo results in this study further demonstrated that the covalently encapsulated DOX exhibited enhanced antitumor efficacy compared with the free DOX. Therefore, as a potential DDS, our covalent conjugation approach provides a promising and novel angle to realize the high drug loading content and stimulus‐controlled drug release behavior in the context of pSiNPs and addresses the critical problem of lack of controlled release behavior that has been plaguing the translation of pSiNPs in nanomedicine.

## 4 Experimental Section

### HPLC Determination of Stimulus‐Mediated SCL Cleavage

The presynthesized SCL‐DOXs was dissolved in DMSO to make the concentration at 10 × 10^−3^ m. For **PCL‐DOX**, 100 µL of stock solution was diluted in 900 µL of PBS and exposed to the photoirradiation (*λ* = 400 nm, 849 mW cm^−2^). 100 µL of reaction mixture was withdrawn at 0, 3, 6, 9, 12, 15, 18, 21, 24, and 27 s and replaced with fresh PBS, samples were then subjected to analytical HPLC. For **ACL‐DOX**, 100 µL of stock solution was diluted in 900 µL of sodium acetate buffer (pH 5.2) or PBS (pH 7.4) and incubated at 37 °C for 24 h. Sample was withdrawn at 0, 0.5, 1, 2, 3, 4, 5, 6, 10, and 24 h and NaHCO_3_ solution was added to adjust pH to neutral, the samples were then analyzed by analytical HPLC (mobile phase: MeCN and (NH_4_)_3_PO_4_ solution). For **ECL‐DOX**, 100 µL of stock solution was diluted in 600 µL of PBS with 3% BSA (w/v), followed by the addition of predissolved *β*‐glucuronidase to make the enzyme concentration at 250 U mL^−1^ (recommended concentration from Sigma‐Aldrich). The solution was aliquoted into seven vials and incubated at 37 °C for 0, 10, 20, 30, 45, 60, and 90 min, respectively. Afterward, glycine buffer solution (1 m, pH 10.0) was added to the reaction mixture and the samples were analyzed by analytical HPLC.

### Evaluation of Mass Loading via Fluorescence Spectroscopy

The presynthesized SCL‐DOXs were dissolved in DMSO to make the concentration at 10 × 10^−3^ m. For **PCL‐DOX**, 100 µL of stock three pSiNP‐SCL‐DOXs at the concentration of 0.06 mg mL^−1^ were exposed to the corresponding stimulus to ensure sufficient cleavage of SCLs and the treated pSiNP solutions were sonicated thoroughly to burst the release of DOX contents from pSiNP. After centrifugation, the supernatant was collected and the DOX concentration was measured by a PerkinElmer EnSpire multimode microplate reader (*λ*_ex/em_ = 470/595 nm). The mass loading of pSiNP‐SCL‐DOX was calculated as follows

(1) $$\mathrm{Mass}\;\;\mathrm{loading}=\frac{W_{1}}{W_{0}}$$

where *W*_1_ is the weight of loaded drugs and *W*_0_ is the weight of pSiNPs.

### Evaluation of Mass Loading via TGA

The TGA was performed on the TA instruments Q50 TGA under a N_2_ gas purge and carbon, hydrogen and nitrogen (C/H/N) elementary analysis (CHNS analyzer, Vario 219 MICRO cube). 1 mg of pSiNP‐SCL‐DOXs/DOX/**pSiNP‐N_3_**/**pSiNP‐UA** in powder was loaded to an empty platinum crucible that was pretreated by annealing in a natural gas flame for ≈30 s. The sample was inserted into the instrument under 50 mL min^−1^ N_2_ atmosphere and left to stabilize for 30 min. The thermal cycle 25–600 °C (10 °C min^−1^) was then initiated maintaining the same N_2_ flow. DOX showed an initial mass loss with a maximum at 100–110 °C that can be assigned to the loss of H_2_O. This is followed by a mass loss that occurs between 200 and 600 °C, which is attributable to the combustion of DOX. Different from DOX, the mass of **pSiNP‐N_3_** underwent a continuous increase over the thermal cycle, owing to the oxidation of pSiNPs. For pSiNP‐SCL‐DOXs comprising of **pSiNP‐N_3_** and DOX, their mass change is affected by the thermal decomposition of DOX and oxidation of pSiNPs simultaneously. At specific temperature (500 °C), the mass loading of pSiNP‐SCL‐DOX can be calculated as follows

(2) $$\mathrm{Mass}\;\;\mathrm{loading}=\frac{\left(W_{\mathrm{initial}}-W_{\mathrm{final}}\right)}{W_{\mathrm{final}}}$$

where *W*_initial_ is the starting weight of pSiNP‐SCL‐DOX and *W*_final_ is the residual weight of pSiNP‐SCL‐DOX.

### In Vitro Release of DOX from pSiNP‐SCL‐DOXs

For control group, pSiNP‐SCL‐DOXs (1 mg) were suspended in 1 mL of PBS and incubated in the dark at 37 °C for 48 h. For experimental group, 1 mg of **pSiNP‐PCL‐DOX**/**pSiNP‐ECL‐DOX** was dispersed in 1 mL PBS, followed by the treatment with photoirradiation (*λ* = 400 nm, 849 mW cm^−2^) for 24 s or *β*‐glucuronidase at a final concentration of 250 U mL^−1^. For 1 mg of **pSiNP‐ACL‐DOX**, 1 mL of sodium acetate buffer (pH 5.2) was added. The treated pSiNP‐SCL‐DOXs were then incubated in the dark at 37 °C. At specific time points over 48 h (0, 1, 2, 4, 6, 8, 10, 24 and 48 h), the particles of both groups were centrifuged and 100 µL of supernatant was removed and replaced with fresh PBS. The DOX concentration in the supernatant was measured by microplate reader (*λ*_ex/em_ = 470/595 nm). The previously obtained drug loading capacity from spectroscopy of each pSiNP‐SCL‐DOX was treated as 100%.

### Cellular Distribution of pSiNP‐SCL‐DOXs

Confocal microscopy was used to investigate the cellular distribution of pSiNP‐SCL‐DOXs. HeLa cells were seeded into glass bottom cell culture dishes at a density of 5000 cells per dish. After 24 h, the medium was replaced with fresh medium. Subsequently, **pSiNP‐ACL‐DOX**, **pSiNP‐PCL‐DOX** (with photoirradiation) and **pSiNP‐ECL‐DOX** (with *β*‐glucuronidase) were treated to the cells at a final concentration of 50 µg mL^−1^ and incubated for 0, 1, 3, 6 and 12 h. After being washed twice with PBS (pH 7.4), the cells were fixed with a 4% paraformaldehyde solution for 15 min at 25 °C. Staining nuclei of incubated cells with 4',6‐diamidino‐2‐phenylindole (DAPI, 3 mmol) were performed for 5 min at 25 °C with several washes in PBS and the intracellular localization of pSiNP‐SCL‐DOXs was observed using a confocal fluorescence microscope (Leica TCS SP8, Leica Microsystems).

### In Vitro Cytotoxicity

HeLa cells (1 × 10^4^) or C32 melanoma cells (2.5 × 10^3^) were seeded into each well of a 96‐well plate. After 24 h, the medium was replaced with fresh medium. Subsequently, the cells were treated with 10–100 µg mL^−1^ of pSiNP‐SCL‐DOXs, including **pSiNP‐PCL‐DOX** (photo irradiated or not), **pSiNP‐ECL‐DOX** (enzyme treated or not), and **pSiNP‐ACL‐DOX**. The cells were also treated with **pSiNP‐N_3_** at the concentration of 50 µg mL^−1^ as control. 48 h later, the cell viability were evaluated via a colorimetric assay using the CellTiter Glo 2.0 Luminescent Cell Viability Assay kit (Promega) following the manufacturer's instructions. All experiments were performed in quadruplicates.

### In Vivo Antitumor Efficiency

Female BALB/c nude mice (4–6 weeks) were purchased from Beijing Vital River Laboratory Animal Technology Co., Ltd. Animal handling procedures were approved by the Animal Ethics Committee of the Northwestern Polytechnical University, China (No. 202001037). The mice were kept under specific pathogen‐free conditions with free access to standard food and water. All animal studies were conducted in accordance with the guidelines of the National Regulation of China for Care and Use of Laboratory Animals. For antitumor efficiency study, female BALB/c nude mice were subcutaneously injected with 1 × 10^6^ HeLa cells in bilateral scapular region. When the tumor volume reached about 50–100 mm^3^ on day 11, the mice were intravenously injected via tail vein one of the follows (*N* = 5): a) PBS, b) **pSiNP‐N_3_** (4 mg kg^−1^), c) **pSiNP‐N_3_** (4 mg kg^−1^) with photoirradiation, d) DOX (1 mg kg^−1^), e) **pSiNP‐PCL‐DOX** (7.3 mg kg^−1^), f) **pSiNP‐PCL‐DOX** (7.3 mg kg^−1^) with photoirradiation, and g) **pSiNP‐ACL‐DOX** (6.1 mg kg^−1^) on day 11, 14, 17, 20, and 23. For radiation‐treated groups, light (*λ* = 400 nm, 849 mW cm^−2^, 24 s) was given 2 d after each particles treatment. The in vivo antitumor efficiency was evaluated by measuring the tumor volume of mice of each group every day. Tumor volume (*V*, mm^3^) was calculated as follows

(3) $$V=\frac{a\times b^{2}}{2}$$

where *a* is the largest and *b* is the smallest diameter. Mice were sacrificed by cervical dislocation on day 24 and the tumor was isolated for weighing test.

### Histological Analysis

HeLa tumor‐bearing mice received the same treatment as described in the in vivo antitumor study. On day 24, the mice were sacrificed. Tumors were collected, fixed in 10% neutral buffered formalin, and embedded in paraffin. The sliced tumors were stained with hematoxylin and eosin (H&E) and visualized by optical microscope. For TUNEL apoptosis assay, the sliced tumor was stained with DAPI and One step TUNEL Apoptosis Assay Kit (Meilunbio) according to the manual. The obtained slides were observed by confocal microscopy as described above.

### Statistical Analysis

Data were presented as means ± SD (standard deviation). Each experiment was performed in triplicate (except for the in vitro and in vivo treatments, which were performed in quadruplicates and quintuples, respectively). The differences between groups were determined using Student's *t*‐test. All statistical tests were two‐sided, and *P*‐values less than 0.1 were considered significant. GraphPad Prism version 9 (GraphPad Software, San Diego, CA) was used for statistical analysis.

## Conflict of Interest

The authors declare no conflict of interest.

## Supporting information

Supporting information

## Data Availability

The data that support the findings of this study are available from the corresponding author upon reasonable request.
