# Supplementary material for: Porous Silicon Nanocarriers with Stimulus‐Cleavable Linkers for Effective Cancer Therapy
Source: Adv Healthc Mater. 2022 Apr 3;11(12):2200076. doi: 10.1002/adhm.202200076 (PMC11468814; doi:10.1002/adhm.202200076)
Supplement: Supplementary file 1 — Supporting information [file ADHM-11-2200076-s001.pdf]

ADVANCED HEALTHCARE MATERIALS

Supporting Information

for *Adv. Healthcare Mater.*, DOI 10.1002/adhm.202200076

Porous Silicon Nanocarriers with Stimulus-Cleavable Linkers for Effective Cancer Therapy

Yufei Xue, Hua Bai, Bo Peng, Terence Tieu, Jiamin Jiang, Shiping Hao, Panpan Li, Mark Richardson, Jonathan Baell, Helmut Thissen, Anna Cifuentes, Lin Li* and Nicolas H. Voelcker**

Supporting Information

Porous silicon nanocarriers with stimulus-cleavable linkers for effective cancer therapy

Yufei Xue[a,b],[†] Hua Bai[a],[†] Bo Peng[a,b],[†],* Terence Tieu[b], Jiamin Jiang[a], Shiping Hao[a], Panpan Li[a], Mark Richardson[c], Jonathan Baell[b], Helmut Thissen[c], Anna Cifuentes[b], Lin Li[a],* Nicolas H. Voelcker[a,b,c,d,e]*

a. Frontiers Science Center for Flexible Electronics, Xi'an Institute of Flexible Electronics (IFE) and Xi'an Institute of Biomedical Materials & Engineering, Northwestern Polytechnical University, 127 West Youyi Road, Xi'an 710072, China.

b. Drug Delivery, Disposition and Dynamics, Monash Institute of Pharmaceutical Sciences, Monash University, Parkville, Victoria 3052, Australia

c. Drug Delivery, Disposition and Dynamics, Monash Institute of Pharmaceutical Sciences, Monash University, Parkville, Victoria 3052, Australia Commonwealth Scientific and Industrial Research Organisation (CSIRO), Clayton, Victoria 3168, Australia

d. Melbourne Centre for Nanofabrication, Victorian Node of the Australian National Fabrication Facility, Clayton, Victoria 3168, Australia

e. Department of Materials Science & Engineering, Monash University, Clayton, Victoria 3168, Australia

[†]Equally contributing first authors

*Correspondence: iambpeng@nwpu.edu.cn, iamlli@nwpu.edu.cn, nicolas.voelcker@monash.edu

EXPERIMENTAL SECTION

Materials and Reagents. All chemicals were purchased from commercial vendors and used without further purification, unless otherwise noted. β-glucuronidase (G7396-25KU) were purchased from Sigma-Aldrich. **PCL-DOX**, **ACL-DOX** and **ECL-DOX** were synthesized as previously reported.[1]

Theoretical Determination of Maximum Loading Capacity

The loading capacity achieved by surface loading ($LC_{surface}$)[2] can be calculated from:

$$LC_{surface} = A_{pSi} \times M_{W(drug)} / A_{drug} \times N_A \quad (S1)$$

where A_{pSi} is the total surface area of respective pSiNPs (489.17 m^2/g),[3] A_{drug} is the minimal projection area of respective drug (7.8×10^{-19} m^2/molecule), N_A is the Avogadro constant (6.022×10^{23}/mol), and $M_{W(drug)}$ is the molecular weight of respective drug (DOX, 543.53 g/mol).

The loading capacity achieved through cavity loading (LC_{cavity})[2] is calculated as follows:

$$LC_{cavity} = V_{pSi} \times \rho_{drug} \quad (S2)$$

where V_{pSi} is the pore volume of the pSiNPs (0.741 cm^3/g),[4] ρ_{drug} is the amorphous density of the loaded drug (DOX, 1.387 g/cm^3).

The apparent density of pSiNPs (ρ_p) is calculated as follows:

$$\rho_p = \rho_{sk} / (\rho_{sk} \times \omega_p + 1) \quad (S3)$$

Where ρ_{sk} is the skeletal density of the pSiNPs (2.33 g/cm^3),[4] ω_p is the porosity of the pSiNPs (0.741 cm^3/g).

The external surface area of pSiNPs ($A_{pSi,\ external}$) is calculated as follows:

$$A_{pSi,\ external} = 4 / (\rho_p \times d) \quad \text{(S4)}$$

Where ρ_p is the apparent density of the pSiNPs (0.853 g/cm^3), d is the average diameter of the pSiNPs (200 nm).

The maximum loading capacity (LC_{max}) of pSiNPs is calculated as follows:

$$LC_{max} = LC_{external\ surface} + LC_{cavity} \quad \text{(S5)}$$

where $LC_{external\ surface}$ is the loading capacity achieved by external surface loading which is calculated by substituting $A_{pSi,\ external}$ (23 m^2/g) into equation 1, LC_{cavity} is the loading capacity achieved through cavity loading.

Cell Culture. HeLa cells were cultured in Dulbecco's modified Eagle's medium (DMEM), supplemented with 4.5 g/L glucose, 10% FBS, 100 U/mL penicillin and 100 µg/mL streptomycin. C32 cells were cultured in Roswell Park Memorial Institute-1640 (RPMI-1640) medium, supplemented with 10% FBS, 100 U/mL penicillin and 100 µg/mL streptomycin. All cells were cultured at 37˚C in an incubator with 5% CO_2 and humidified atmosphere. Cell viability was measured *via* a colorimetric assay using the CellTiter Glo® 2.0 Luminescent Cell Viability Assay kit (Promega, G9241).

Characterization. Liquid chromatography-Mass spectrometry (LC-MS) was performed using an Agilent UHPLC/MS 1260/6120 with a 1260 Infinity G1312B Binary pump and a

1260 Infinity G1367E 1260 HiP ALS autosampler. The detector used is a 1290 Infinity G4212A 1290 DAD, which monitors at 254 nm and 214 nm. High performance liquid chromatography (HPLC) was completed using reverse phase HPLC analysis with a Poroshell 120 EC-C18 column. Mass spectrometry was performed using performed using a Waters LCT TOF LC/MS Mass Spectrometer coupled to a 2795 Alliance Separations module. All 1H NMR and ^{13}C NMR spectra were taken on a Avance III Nanobay 400 MHz Bruker spectrometer, using $CDCl_3$ or $(CD_3)_2SO$ as the solvent. Chemical shifts are reported in parts per million referenced with respect to residual solvent ($CDCl_3$ = 7.26 ppm, and $(CD_3)_2SO$ = 2.50 ppm) for ^1H NMR, ($CDCl_3$ = 77.16 ppm and $(CD_3)_2SO$ = 39.52 ppm) for ^{13}C NMR. Extent of reaction was monitored by thin layer chromatography (TLC) using Merck 60 F254 pre-coated silica gel plates with fluorescent indicator UV254. After the plates were subjected to elution in the TLC chamber, the spots were visualized under UV light or using the appropriate stain (I_2, $KMnO_4$). Transmission electron microscopy (TEM) images were obtained from a JEOL JEM-2100F with an accelerating voltage of 200 kV. Particle size and zeta potential of the nanoparticles were measured by dynamic laser-light scattering (DLS) using a Zetasizer Nano ZS. Fourier-transform infrared spectroscopy (FTIR) was conducted on a Hyperion 1000 FTIR microscope coupled to a Vertex 70 IR source (Bruker, Germany) and a liquid-N_2-cooled MCT detector. Thermogravimetric analysis (TGA) was performed on the TA instruments Q50 TGA under a N_2 gas purge and CHN elementary analysis (CHNS analyser, Vario 219 MICRO cube). The HeLa cells were imaged by confocal laser scanning microscope (Leica TCS SP8, Leica Microsystems). Cell viability assays were measured by a PerkinElmer EnSpire multimode microplate reader. H&E and

TUNEL images were taken using an Axiovert imager M1 fluorescence microscope (Zeiss, Oberkochen,Germany).

Synthesis of PCL-DOX

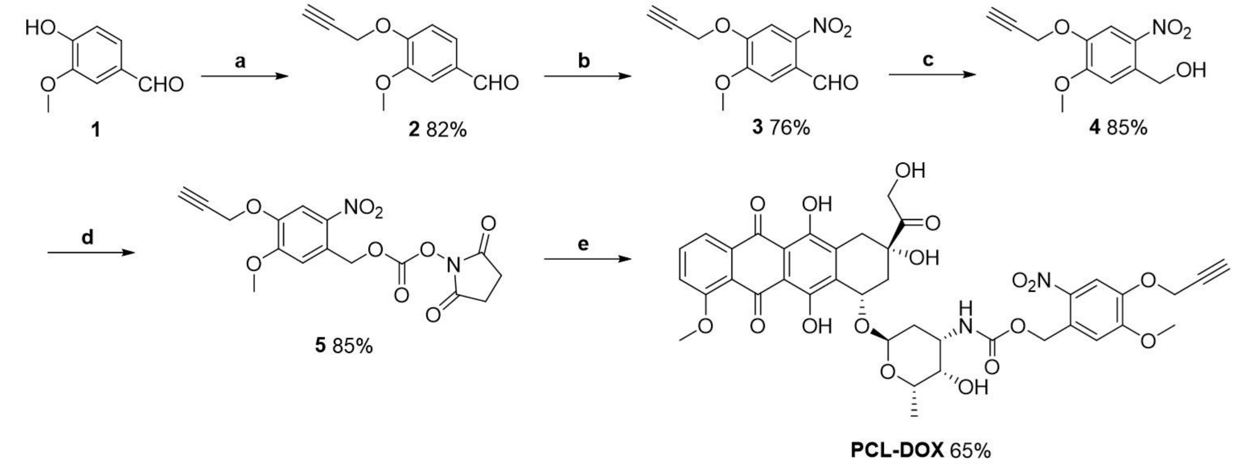

Scheme S1. Synthetic route of photo-cleavable linker-DOX conjugate (**PCL-DOX**). Conditions: (a) 3-bromo-1-propyne, Cs_2CO_3, DMF, room temperature, 24 h; (b) HNO_3, 0°C, 1 h; (c) $NaBH_4$, EtOH, room temperature, 2.5 h; (d) *N, N'*-disuccinimidyl carbonate, dry MeCN, Et_3N, room temperature, 1 h; (e) DOX, DIPEA, dry DMF, room temperature, 1 h.

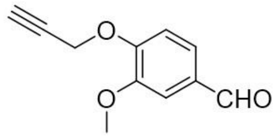

(2)

Vanillin (**1**) (1.5 g, 9.86 mmol) was stirred with Cs_2CO_3 (3.21 g, 9.86 mmol) for 5 min in dry *N, N*-Dimethylformamide (DMF, 25 mL). To the mixture was added 3-bromo-1-propyne (3.74 mL, 49.3 mmol). The reaction was stirred at room temperature for 24 h. The reaction was then quenched with H_2O (30 mL) and extracted with ethyl acetate (EA) (3 × 30 mL) and back extracted with brine (3 × 80 mL). The organic layer was dried over $MgSO_4$, filtered, and concentrated by rotatory evaporation. The resulting solution

was purified via flash chromatography (EA : Hexane = 1 : 3), resulting in a white solid (1.52 g, 81%). ^{1}H NMR (400 MHz, $CDCl_3$) δ 9.87 (s, 1H), 7.46 (dd, *J* = 8.2, 1.9 Hz, 1H), 7.43 (d, *J* = 1.8 Hz, 1H), 7.14 (d, *J* = 8.2 Hz, 1H), 4.86 (d, *J* = 2.4 Hz, 2H), 3.94 (s, 3H), 2.56 (t, *J* = 2.4 Hz, 1H). DEPT NMR (101 MHz, $CDCl_3$) δ 190.84, 152.12, 150.05, 130.94, 126.18, 112.66, 109.54, 77.49, 76.67, 56.60, 56.01. LC-MS (ESI) calcd for $[M+H]^+$: 191.06, found 191.10.

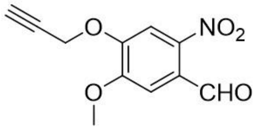

(3)

A flask was charged with **2** (1.5 g, 8.00 mmol), wrapped in aluminium foil and incubated on ice. Chilled HNO_3 (50 mL, excess) was added to the flask and allowed to stir for 25 min at 0°C. The reaction was then warmed to room temperature for 2 h. The reaction was quenched with chilled H_2O (100 mL) and the precipitate was collected by vacuum filtration and washed with ice H_2O (3 × 30 mL). The resulting was a yellow solid (1.53 g, 82%) and required no further purification. ^{1}H NMR (400 MHz, $CDCl_3$) δ 10.45 (s, 1H), 7.79 (s, 1H), 7.43 (s, 1H), 4.91 (d, *J* = 2.4 Hz, 2H), 4.02 (s, 3H), 2.63 (t, *J* = 2.4 Hz, 1H). DEPT NMR (101 MHz, $CDCl_3$) δ 187.67, 153.75, 149.94, 143.41, 126.44, 110.28, 109.48, 76.72, 76.39, 57.25, 56.78. LC-MS (ESI) calcd for $[M+H]^+$: 236.05, found 236.03.

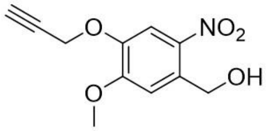

(4)

Compound **3** (1.53 g, 6.52 mmol) was dissolved in ethanol (EtOH, 120 mL) and wrapped in aluminium foil. $NaBH_4$ (740 mg, 19.6 mmol) was dissolved in NaOH (50 mL,

1 M). The resulting solution was then added to **3** and allowed to stir at room temperature for 2.5 h. The reaction was neutralized by 1 M HCl, extracted with EA (3 × 30 mL) and back-extracted with brine (3 × 70 mL). The organic layer was dried with $MgSO_4$ and concentrated using a rotary evaporator. The resulting compound was a pale-yellow solid (1.78 g, 76%) and required no further purification. ^{1}H NMR (400 MHz, $CDCl_3$) δ 7.89 (s, 1H), 7.22 (s, 1H), 4.98 (s, 2H), 4.84 (d, *J* = 2.4 Hz, 2H), 4.01 (s, 3H), 2.58 (t, *J* = 2.4 Hz, 1H). DEPT NMR (101 MHz, $CDCl_3$) δ 154.53, 133.36, 111.25, 76.98, 62.78, 57.11, 56.49. LC-MS (ESI) calcd for $[M+Na]^+$: 260.06, found 259.93.

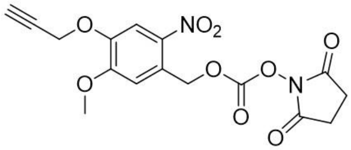

(5)

To a solution of **4** (200 mg, 0.79 mmol) dissolved in acetonitrile (MeCN, 3 mL), triethylamine (Et_3N, 151 mg, 1.5 mmol) and *N, N'*-disuccinimidyl carbonate (220 mg, 0.95 mmol) were added. After being stirred for 1.5 h under N_2 at room temperature, TLC analysis showed that the starting material was completely consumed. The solvent was removed, and the residue was purified by flash column chromatography (EA : Hexane = 1 : 2) to provide the desired product as a light yellow solid (250 mg, 85%). ^{1}H NMR (400 MHz, $CDCl_3$) δ 7.95 (s, 1H), 7.08 (s, 1H), 5.80 (d, *J* = 0.6 Hz, 2H), 4.85 (d, *J* = 2.4 Hz, 2H), 4.06 (s, 3H), 2.86 (s, 4H), 2.59 (s, 1H). ^{13}C NMR (101 MHz, $CDCl_3$) δ 168.36, 154.81, 151.42, 146.11, 126.50, 111.13, 109.06, 69.09, 57.12, 56.69, 30.88, 25.46. LC-MS (ESI) calcd for $[M+Na]^+$: 401.07, found 400.94.

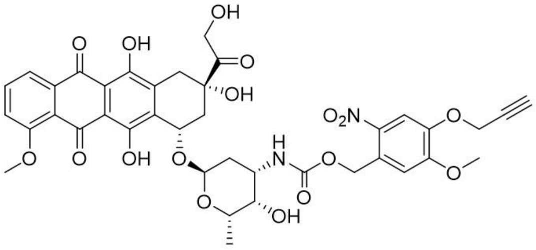

PCL-DOX

Compound **5** (10 mg, 0.026 mmol) in anhydrous DMF (3 mL), was added DOX (13 mg, 0.024 mmol) and *N, N*-diisopropylethylaime (DIPEA, 6.72mg, 0.052 mmol) at room temperature. The reaction was stirred under an atmosphere of N_2 for 1 h and LC-MS indicated full consumption of the **5** with the formation of the final product **PCL-DOX**. Reaction mixture was diluted with EA (10 mL) and washed with H_2O (3 × 10 mL) and brine (2 × 10 mL), followed by purification with silica gel flash column chromatography (dichloromethane (DCM) : methanol (MeOH) = 20 : 1) to give the desired product as a red solid (13.6 mg, 65%). ^{1}H NMR (401 MHz, $CDCl_3$) δ 8.02 (d, *J* = 7.1 Hz, 1H), 7.88-7.72 (m, 2H), 7.44-7.34 (m, 1H), 6.96 (s, 1H), 5.54-5.41 (m, 3H), 5.32-5.25 (m, 4H), 4.86-4.71 (m, 4H), 4.13 (dd, *J* = 14.5, 7.2 Hz, 1H), 4.07 (s, 2H), 3.94 (s, 2H), 3.69 (s, 1H), 3.48 (s, 1H), 3.25 (ddd, *J* = 18.6, 9.7, 1.8 Hz, 1H), 3.00 (d, *J* = 18.9 Hz, 1H), 2.55 (t, *J* = 2.3 Hz, 1H), 2.33 (d, *J* = 14.8 Hz, 1H), 2.17 (dd, *J* = 14.7, 4.0 Hz, 1H), 1.90 (dd, *J* = 13.3, 4.6 Hz, 1H), 1.84-1.75 (m, 1H), 1.29 (d, *J* = 6.6 Hz, 2H). ^{13}C NMR (101 MHz, $CDCl_3$) δ 186.70, 161.08, 155.63, 145.67, 135.94, 135.81, 135.50, 133.58, 119.89, 118.49, 111.45, 110.97, 110.58, 100.64, 77.33, 76.61, 69.77, 69.57, 67.22, 65.55, 63.59, 62.19, 57.03, 56.68, 56.47, 53.41, 50.87, 35.63, 33.94, 30.21. HRMS (ESI) calcd for $[M+Na]^+$: 829.2170, found 829.2053.

Synthesis of ACL-DOX

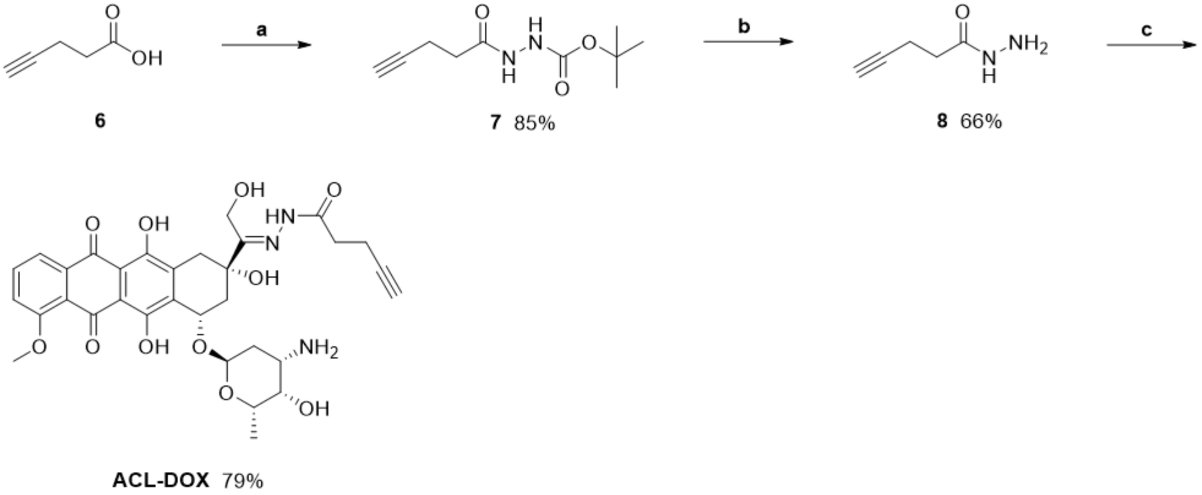

Scheme S2. Synthetic route of acid-cleavable linker-DOX conjugate (ACL-DOX). Conditions: (a) Tert-butyl carbazate, EDCI·HCl, HOBt, dry DCM, room temperature, 16 h; (b) 4M HCl in dioxane, room temperature, 1 h; (c) DOX, AcOH, Na_2SO_4, dry MeOH, room temperature, 20 h.

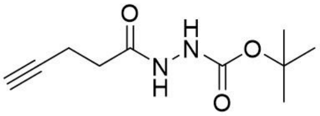 **(7)**

To a mixture of 4-pentynoic acid (**6**) (49 mg, 0.5 mmol) and EDCI·HCl (115 mg, 0.6 mmol) in 5 mL of dry DCM was added HOBt (81 mg, 0.6 mmol) and the reaction was allowed to stir for 30 min under N_2 atmosphere. Tert-butyl carbazate (66 mg, 0.5 mmol) was then added and the reaction vessel was stirred in a N_2 atmosphere for 18 h. The reaction was tracked via TLC using potassium permanganate as a TLC stain. The solvent was concentrated under vacuum to give a white solid which was purified using

silica gel chromatography with a gradient of 1-5% in MeOH of a DCM : MeOH solution in increments of 100 mL to yield a pale white solid (90 mg, 85%). ^{1}H NMR (400 MHz, $CDCl_3$) δ 7.75 (s, 1H), 6.68 (s, 1H), 2.65-2.38 (m, 5H), 1.99 (dt, J = 14.5, 2.6 Hz, 1H), 1.47 (s, 9H). ^{13}C NMR (101 MHz, $CDCl_3$) δ 170.72, 155.74, 82.68, 82.30, 69.80, 33.24, 28.38, 14.69.

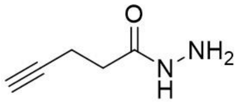

(8)

To **7** (30 mg, 0.14 mmol) was added 1.5 mL of 4 N HCl in dioxane. After being stirred for 1 h at room temperature, TLC analysis showed the starting material was completely consumed. The solvent was removed under vacuum to yield a pale-yellow solid used without further purification (10.5 mg, 66%).

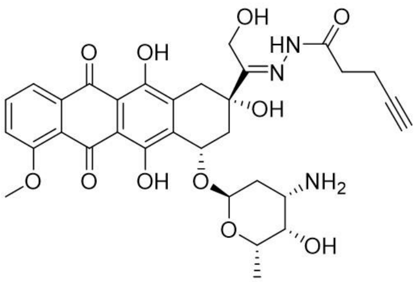

ACL-DOX

Compound **8** (10 mg, 0.089 mmol) and Na_2SO_4 (12.6 mg, 0.089 mmol) were dissolved in 5 mL of dry MeOH in a round bottom flask at room temperature under N_2 atmosphere. To this was added DOX (48.5 mg, 0.089 mmol) and a drop of acetic acid (AcOH). The reaction was allowed to stir for 24 h. After the reaction was completed, Na_2SO_4 was filtered out and the filtrate was concentrated under vacuum to form a red oily solid. The red solid was diluted in 5 mL of dry MeCN and the precipitate was collected by filtration and washed with dry MeCN (3 × 5 mL) to yield 45 mg (79.4%) **ACL-DOX** product. ^{1}H

NMR (400 MHz, DMSO-d_6) δ 5.86 (s, 1H), 5.51 (s, 1H), 5.43 (s, 2H), 5.33-5.25 (m, 1H), 4.95-4.90 (m, 1H), 4.59 (s, 1H), 4.43 (s, 1H), 4.13 (dd, *J* = 43.5, 6.8 Hz, 1H), 3.97 (d, *J* = 2.6 Hz, 3H), 3.61 (s, 1H), 3.36 (d, *J* = 11.3 Hz, 2H), 3.03-2.88 (m, 1H), 2.80 (t, *J* = 2.4 Hz, 1H), 2.43-2.35 (m, 4H), 2.29-2.06 (m, 2H), 1.89 (dd, *J* = 16.5, 7.9 Hz, 1H), 1.70 (dd, *J* = 19.6, 7.7 Hz, 1H), 1.16 (dd, *J* = 9.4, 6.6 Hz, 3H). ^{13}C NMR (101 MHz, DMSO-d_6) δ 186.97, 172.65, 170.31, 161.24, 156.88, 154.74, 153.88, 136.71, 135.26, 120.51, 119.46, 111.11, 99.48, 83.98, 72.60, 72.26, 72.12, 71.38, 66.76, 66.46, 57.08, 56.55, 47.06, 32.29, 31.18, 17.30, 14.16, 13.45. HRMS (ESI) calcd for $[M+H]^+$: 638.2272, found 638.2357.

Synthesis of ECL-DOX

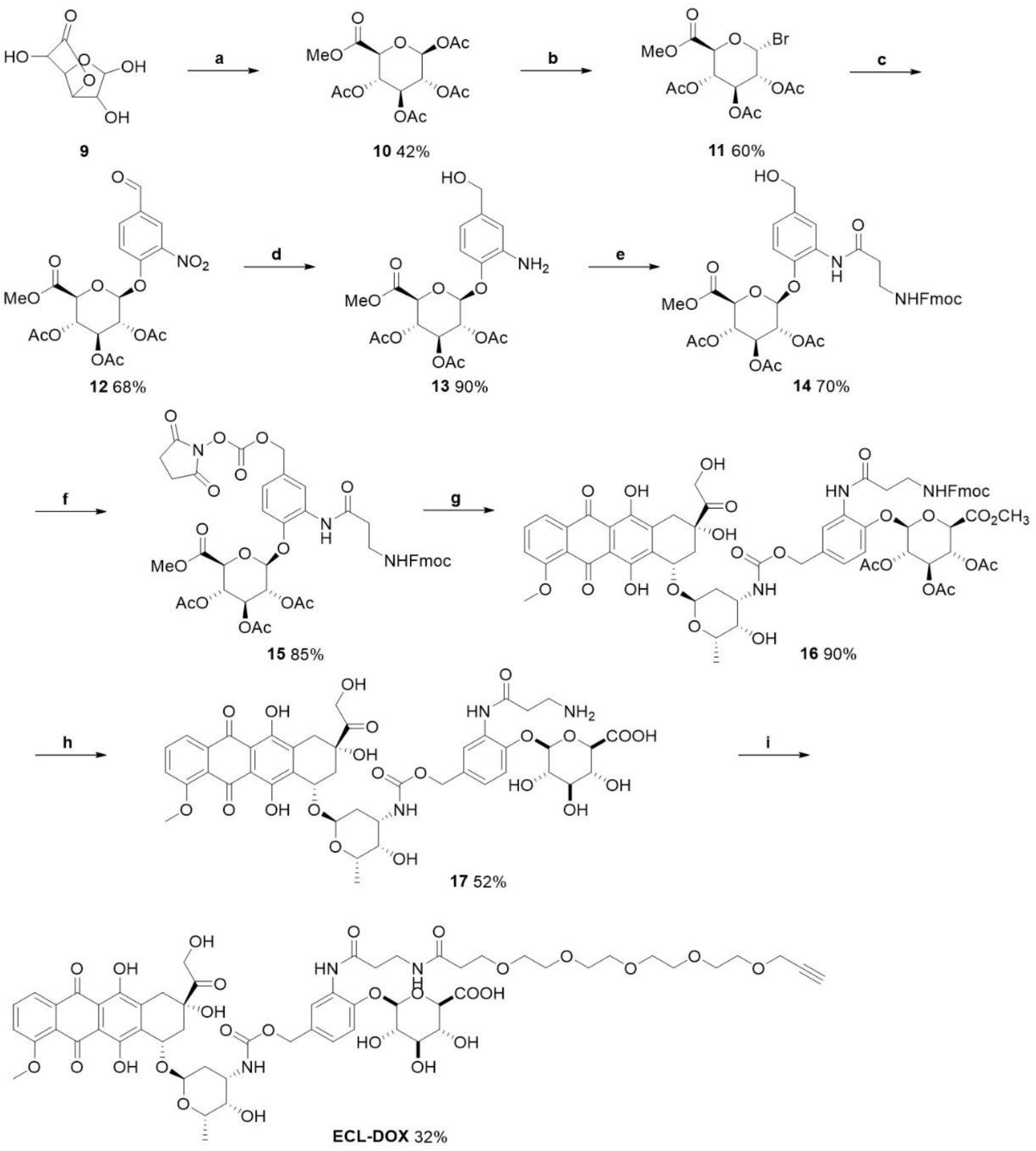

Scheme S3. Synthetic route of enzyme-cleavable linker-DOX conjugate (**ECL-DOX**). Conditions: (a) *N, N*-dimethylethylamine, MeOH, room temperature, 5 h; Ac_2O, pyridine, room temperature, overnight; (b) HBr, DCM, 0°C, 4 h; (c) 4-hydroxy-3-nitrobenzaldehyde, Ag_2O, dry MeCN, room temperature, 4 h; (d) $NaBH_4$, THF, 0°C, 2 h; Fe, AcOH, EtOH : H_2O (4 : 1), reflux, 1 h; (e) Fmoc-β-alanine, HATU, DIPEA, dry DMF, 0°C to room temperature, 3 h; (f) *N, N'*-disuccinimidyl carbonate, Et_3N, dry MeCN, room temperature, 2 h; (g) DOX, DIPEA, dry DMF, room temperature, 2 h; (h) LiOH

(1M in H_2O), MeOH, 0°C, 35 min; piperidine, DMF, room temperature, 5 min; (i) alkyne-PEG4-ester, DIPEA, dry DMF, room temperature, 1.5 h.

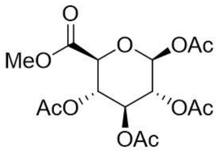

(10)

D (+)-Glucurono-6,3-lactone (**9**) (5 g, 28.4 mmol) was suspended in dry MeOH (80 mL), to this *N, N*-dimethylethylamine (0.05 mL) was added. The reaction was stirred for 5 h until all the glucuronolactone was dissolved. The solvent was removed under reduced pressure, and the foam was used without purification. Acetic anhydride (Ac_2O, 25 mL) and pyridine (37 mL) were added and the suspension was stirred overnight. TLC indicated that the D (+)-glucuronic acid was fully consumed and the solvent was removed under reduced pressure and recrystallized from absolute EtOH to give the title product as a white prism (3.40 g, 42%). ^{1}H NMR (400 MHz, $CDCl_3$) δ 6.88 (d, *J* = 8.2 Hz, 1H), 6.71 (d, *J* = 2.0 Hz, 1H), 6.68-6.58 (m, 1H), 5.37-5.23 (m, 3H), 5.00 (d, *J* = 7.3 Hz, 1H), 4.52 (s, 2H), 4.13 (dd, *J* = 14.7, 7.8 Hz, 1H), 3.75 (d, *J* = 5.3 Hz, 3H), 2.09-2.06 (m, 3H), 2.04 (d, *J* = 3.3 Hz, 6H). ^{13}C NMR (101 MHz, $CDCl_3$) δ 169.87, 169.38, 169.15, 168.80, 166.79, 91.36, 72.99, 71.82, 70.16, 68.91, 52.99, 20.74, 20.52, 20.44.

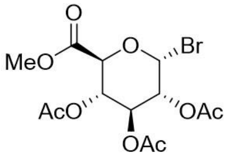

(11)

To **10** (2.0 g, 5.31 mmol) in DCM (5 mL), HBr (33% in AcOH, 8 mL) was added at 0°C and the reaction was stirred for additional 4 h (monitored by TLC). The reaction mixture was diluted with EA (15 mL), washed with H_2O (3 × 10 mL), $NaHCO_3$ (2 × 10 mL), H_2O

(2 × 10 mL), brine (2 × 10 mL), dried over $MgSO_4$, and the solvent was removed under reduced pressure. Recrystallization of the residue from absolute EtOH gave the title compound (1.02 g, 60%) as a white solid. ^{1}H NMR (400 MHz, $CDCl_3$) δ 7.27 (d, J = 4.0 Hz, 1H), 6.24 (t, J = 9.7 Hz, 1H), 5.87 (dd, J = 10.2, 9.6 Hz, 1H), 5.48 (dd, J = 10.0, 4.1 Hz, 1H), 5.21 (d, J = 10.3 Hz, 1H), 4.39 (s, 3H), 2.72 (s, 3H), 2.68 (d, J = 2.5 Hz, 6H). ^{13}C NMR (101 MHz, $CDCl_3$) δ 169.38, 169.15, 168.80, 166.80, 91.38, 73.01, 71.84, 70.19, 68.93, 52.98, 20.73, 20.53, 20.43.

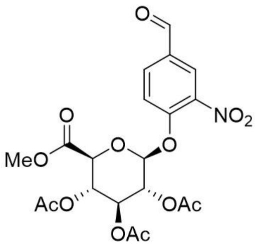

(12)

To a mixture of **11** (1.02 g, 2.57 mmol) in dry MeCN (25 mL) was added 4-hydroxy-3-nitrobenzaldehyde (0.85 g, 5 mmol) followed by addition of silver (I) oxide (Ag_2O, 3.47 g, 15 mmol). The resulting slurry was stirred in the dark under N_2 for 4 h. The solution passed through a pad of Celite to remove Ag_2O and the filtrate concentrated under reduced pressure. The residue was brought up in EA (20 mL) and washed with $NaHCO_3$ (3 × 10 mL), H_2O (3 × 10 mL) and brine (2 × 10 mL). The organic layer was dried over $MgSO_4$, filtered and concentrated under reduced pressure to yield the title product (0.85 g, 68%). ^{1}H NMR (400 MHz, $CDCl_3$) δ 9.98 (s, 1H), 8.31 (d, J = 2.0 Hz, 1H), 8.09 (dd, J = 8.6, 2.1 Hz, 1H), 7.50 (d, J = 8.6 Hz, 1H), 5.47-5.39 (m, 2H), 5.36-5.26 (m, 2H), 4.32 (d, J = 8.4 Hz, 1H), 3.71 (s, 3H), 2.13 (s, 3H), 2.08 (d, J = 4.0 Hz, 6H).^{13}C NMR (101 MHz, $CDCl_3$) δ 188.55, 169.90, 169.23, 169.08, 166.64, 153.28,

134.23, 131.47, 126.69, 118.77, 98.56, 72.68, 70.18, 69.76, 68.12, 53.08, 20.54. LC-MS (ESI) calcd for $[M+Na]^+$: 506.10, found 505.93.

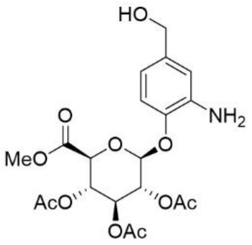

(13)

$NaBH_4$ (117.3 mg, 3.1 mmol) was added to a solution of **12** (500 mg, 1.05 mmol) in THF (20 mL) at 0°C and stirred for 2 h under N_2. The solvent was removed under reduced pressure and saturated NH_4Cl solution (10 mL) was added. The mixture was extracted with EA (3 × 10 mL) and the combined organic layer were washed with H_2O (3 × 10 mL), brine (2 × 10 mL), and dried under reduced pressure to give the desired product as a white solid, which was subsequently re-dissolved in 12.5 mL of a mixed solution (EtOH : H_2O = 4 : 1), followed by addition of iron powder (Fe, 1.13 g, 103.5 mmol) and HCl (3.77 mg, 0.10 mmol). The reaction was allowed to be refluxed under N_2 for 1 h. Removal of solvent gave a white crude product which was further purified through silica gel flash column chromatography (DCM : MeOH = 100 : 1) to provide the title compound (42.2 mg) in a yield of 90%. ^{1}H NMR (400 MHz, $CDCl_3$) δ 6.90 (d, *J* = 8.2 Hz, 1H), 6.73 (d, *J* = 2.0 Hz, 1H), 6.65 (dd, *J* = 8.2, 2.0 Hz, 1H), 5.52-5.14 (m, 3H), 5.02 (d, *J* = 7.2 Hz, 1H), 4.55 (d, *J* = 5.5 Hz, 2H), 4.15 (d, *J* = 9.4 Hz, 1H), 3.85 (s, 2H), 3.75 (s, 3H), 2.08 (s, 3H), 2.05 (d, *J* = 4.2 Hz, 6H). ^{13}C NMR (101 MHz, $CDCl_3$) δ 170.01, 169.65, 169.39, 166.83, 143.72, 137.89, 137.40, 116.66, 114.51, 100.60, 72.56, 71.68, 71.02, 69.29, 64.96, 52.98, 20.73, 20.58, 20.46. LC-MS (ESI) calcd for $[M+H]^+$: 456.14, found 456.21.

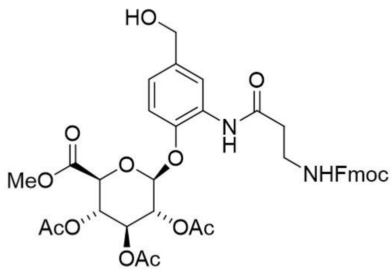

(14)

The Fmoc-β-alanine (75 mg, 0.24 mmol) was dissolved in dry DMF (2 mL) in ice bath. To this was added HATU (92 mg, 0.24 mmol) in three times and the reaction was stirred for 30 min until a homogenous phase was formed. Then the mixture was added to a solution of **13** (74 mg, 0.163 mmol) in dry DMF (5 mL) dropwise at 0°C, followed by addition of DIPEA (7.37 mg, 0.33 mmol). After being stirred under N_2 for 2 h, the reaction mixture was diluted with EA (10 mL) and washed with H_2O (2 × 10 mL) and brine (2 × 10 mL), followed by purification with silica gel flash column chromatography (DCM : MeOH = 50 : 1) to yield the title compound (85 mg, 70%). ^{1}H NMR (400 MHz, $CDCl_3$) δ 8.35 (s, 1H), 8.07 (s, 1H), 7.75 (d, *J* = 7.5 Hz, 2H), 7.59 (d, *J* = 7.4 Hz, 2H), 7.38 (t, *J* = 7.4 Hz, 2H), 7.32-7.25 (m, 2H), 7.07 (dd, *J* = 8.3, 1.5 Hz, 1H), 6.94 (d, *J* = 8.3 Hz, 1H), 5.68 (s, 1H), 5.40 (t, *J* = 9.4 Hz, 1H), 5.35-5.23 (m, 3H), 5.04 (d, *J* = 7.5 Hz, 1H), 4.64 (s, 2H), 4.38 (dd, *J* = 16.2, 8.8 Hz, 2H), 4.22 (t, *J* = 7.0 Hz, 1H), 4.16 (d, *J* = 9.6 Hz, 1H), 3.72 (s, 3H), 3.59 (d, *J* = 4.4 Hz, 2H), 2.72 (s, 2H), 2.05 (t, *J* = 4.4 Hz, 9H). ^{13}C NMR (101 MHz, $CDCl_3$) δ 170.13, 169.81, 169.37, 166.72, 144.62, 144.03, 141.27, 137.40, 127.63, 127.04, 125.14, 122.59, 119.93, 119.56, 100.54, 72.48, 71.14, 69.21, 66.84, 64.86, 53.15, 47.24, 36.80, 20.75, 20.52, 20.43. LC-MS (ESI) calcd for $[M+Na]^+$: 749.25, found 748.94.

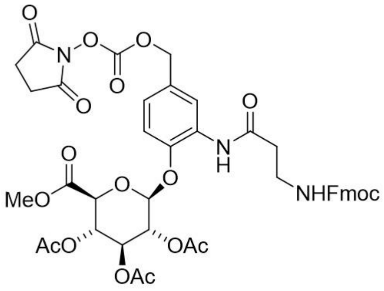

(15)

Compound **15** was synthesized using the same reaction procedure of **5** using *N, N'*-disuccinimidyl carbonate (15.37 mg, 0.06 mmol), Et_3N (6.06 mg, 0.06 mmol) and dry MeCN (5 mL). The crude product **15** was purified by silica gel flash column chromatography (DCM : MeOH = 80 : 1). The title compound was isolated as a white solid (30.23 mg, 85%). ^1H NMR (400 MHz, $CDCl_3$) δ 8.34 (s, 1H), 8.08 (s, 1H), 7.75 (d, *J* = 7.5 Hz, 2H), 7.59 (d, *J* = 7.3 Hz, 2H), 7.38 (t, *J* = 7.4 Hz, 2H), 7.31-7.25 (m, 3H), 7.07 (dd, *J* = 8.3, 1.9 Hz, 1H), 6.94 (d, *J* = 8.3 Hz, 1H), 5.67 (s, 1H), 5.39 (d, *J* = 9.4 Hz, 1H), 5.34-5.24 (m, 2H), 5.04 (d, *J* = 7.5 Hz, 1H), 4.64 (s, 2H), 4.37 (t, *J* = 6.9 Hz, 2H), 4.23 (d, *J* = 6.8 Hz, 1H), 4.15 (d, *J* = 9.6 Hz, 1H), 3.72 (s, 3H), 3.59 (d, *J* = 5.2 Hz, 2H), 2.72 (s, 1H), 2.04 (dd, *J* = 5.8, 4.2 Hz, 9H). ^{13}C NMR (101 MHz, $CDCl_3$) δ 170.29, 169.78, 169.37, 168.70, 168.57, 166.64, 156.46, 151.52, 145.56, 144.03, 141.29, 129.41, 127.63, 127.05, 125.16, 124.32, 121.18, 119.92, 100.10, 72.52, 72.35, 71.20, 70.99, 69.18, 66.82, 53.16, 47.24, 36.96, 25.45, 20.78, 20.52, 20.43. LC-MS (ESI) calcd for $[M+H]^+$: 890.25, found 889.90.

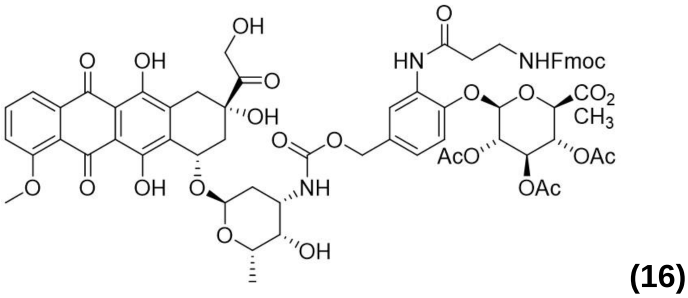

(16)

Compound **15** was synthesized using the same reaction procedure of **PCL-DOX** using DOX (23.60 mg, 0.04 mmol), DIPEA (3.30 mg, 0.05 mmol) and dry DMF (5 mL). The crude product was purified via prep-HPLC (10% organic for 5 min followed by a ramp up to 100% over 30 min) to give the title compound as a red solid (39.9 mg, 90%). ^{1}H NMR (400 MHz, MeOD-d_4) δ 8.14 (s, 1H), 7.99 (s, 1H), 7.86 (s, 1H), 7.72 (dd, *J* = 19.9, 7.7 Hz, 2H), 7.57 (d, *J* = 7.1 Hz, 1H), 7.43 (d, *J* = 8.6 Hz, 1H), 7.31 (t, *J* = 7.4 Hz, 1H), 7.25-7.18 (m, 1H), 7.03 (dd, *J* = 20.3, 8.2 Hz, 1H), 6.80 (d, *J* = 8.0 Hz, 1H), 5.43 (d, *J* = 7.0 Hz, 1H), 5.31 (d, *J* = 7.5 Hz, 1H), 5.24-5.11 (m, 2H), 4.76 (d, *J* = 1.8 Hz, 1H), 4.45-4.24 (m, 2H), 4.15 (d, *J* = 6.6 Hz, 1H), 3.97 (s, 1H), 3.65 (d, *J* = 9.2 Hz, 2H), 3.54-3.43 (m, 1H), 3.19-3.00 (m, 2H), 2.62 (s, 1H), 2.39 (d, *J* = 13.2 Hz, 1H), 2.19 (d, *J* = 9.4 Hz, 1H), 2.00 (dd, *J* = 11.5, 4.1 Hz, 9H), 1.79 (d, *J* = 8.9 Hz, 1H), 1.28 (d, *J* = 6.5 Hz, 1H). LC-MS (ESI) calcd for $[M+Na]^+$: 1340.40, found 1340.63.

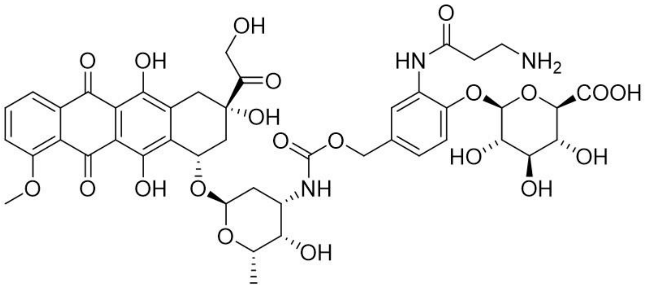

(17)

16 (39.9 mg, 0.03 mmol) was dissolved in 5 mL MeOH at 0°C, to this reaction mixture was added a solution of LiOH (12.6 mg, 0.30 mmol) in H_2O (3 mL). The mixture was

stirred for 35 min and as neutralized with AcOH (17.4 µL, 0.3 mmol) to pH 7. The reaction mixture was concentrated under vacuum to give a residue which was dissolved in DMF (5 mL) and treated with piperidine (1 mL). After being stirred for 5 min, the mixture was concentrated under vacuum to give the crude product that was purified using prep-HPLC (10% organic for 3 min followed by a ramp up to 50% over 25 min) to yield 15 mg (52.4%) of **17** as a red solid. LC-MS (ESI) calcd for $[M+H]^+$: 956.29, found 956.32.

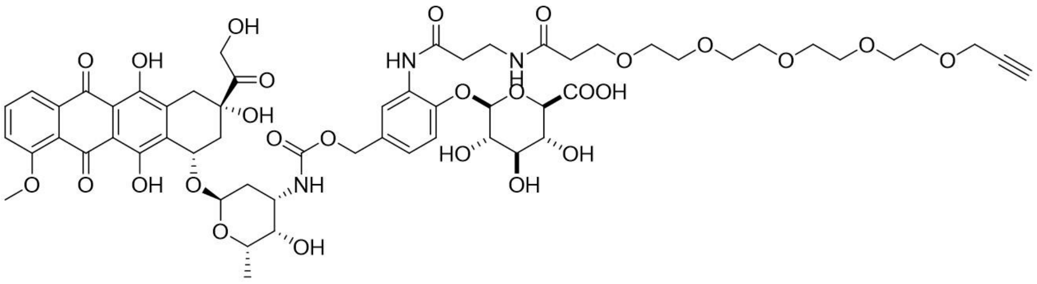

ECL-DOX

Alkyne-PEG4-NHS ester (6.5 mg, 0.016 mmol) and DIPEA (3 µL) were added over 5 min to a solution of compound **17** (15 mg, 0.016 mmol) in dry DMF (3 mL) under N_2 atmosphere. After 30 min, solvent was removed in vacuum to give out a crude product which was further purified via prep-HPLC (10% organic for 3 min followed by a ramp up to 50% over 25 min), yielding 6.5 mg (32.7%) of compound **ECL-DOX** as a red solid. ^1H NMR (400 MHz, DMSO-d_6) δ 9.14 (s, 1H), 8.12 (s, 1H), 8.01 (s, 1H), 7.94 (s, 1H), 7.70 (dd, *J* = 14.8, 3.5 Hz, 1H), 7.07 (d, *J* = 8.1 Hz, 1H), 6.99 (d, *J* = 8.0 Hz, 1H), 6.83 (d, *J* = 7.9 Hz, 1H), 5.73 (s, 1H), 5.49 (s, 1H), 5.36-5.30 (m, 1H), 5.20 (d, *J* = 24.5 Hz, 1H), 4.97 (s, 1H), 4.86 (d, *J* = 11.4 Hz, 1H), 4.74-4.65 (m, 1H), 4.58 (d, *J* = 4.7 Hz, 1H), 4.24 (dd, *J* = 20.8, 4.2 Hz, 1H), 4.14 (d, *J* = 2.4 Hz, 2H), 4.00 (s, 2H), 3.71 (d, *J* = 13.1 Hz, 1H), 3.64 (d, *J* = 4.2 Hz, 1H), 3.58 (d, *J* = 4.4 Hz, 2H), 3.57-3.44 (m, 14H), 3.18 (s, 1H), 3.00 (s, 1H), 2.47-2.39 (m, 1H), 2.30 (t, *J* = 6.4 Hz, 2H), 2.21-2.09 (m, 1H), 1.99 (d, *J* = 6.2

Hz, 1H), 1.84 (dd, J = 12.8, 9.0 Hz, 1H), 1.13 (d, J = 6.5 Hz, 2H). ^{13}C NMR (101 MHz, DMSO-d_6) δ 173.62, 169.48, 169.14, 155.96, 147.77, 110.90, 103.47, 73.03, 69.69, 62.75, 46.55, 14.02. HRMS (ESI) calcd for $[M+Na]^+$: 1264.4275, found 1264.4123.

Synthesis of pSiNP-SCL-DOX

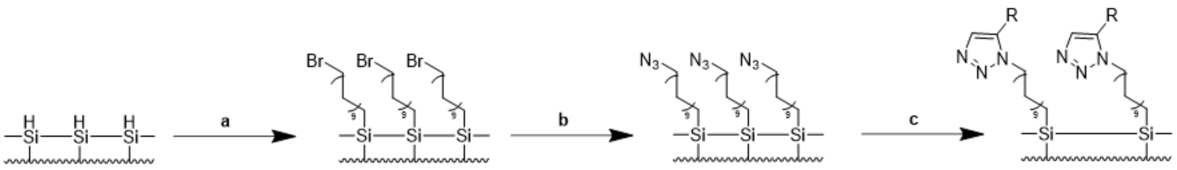

R=PCL-DOX, ECL-DOX or ACL-DOX

Scheme S4. Synthetic route of pSiNP-SCL-DOX. Conditions: (a) 11-bromo-1-undecene, 95°C, 24 h; (b) NaN_3, DMF, 60°C, 24 h; (c) SCL-DOXs, $CuSO_4$, THPTA, Sodium ascorbate, DMSO : H_2O = 1 : 1, room temperature, 24 h.

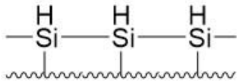

Freshly etched pSiNP

pSi films were prepared by periodically etching p^+ type (0.0055-0.001 Ω cm) silicon wafers at 5 mA/cm^2 for 20 s and 139 mA/cm^2 for 0.2 s for 1000 cycles in a 3 : 1 HF (49%) : EtOH solution. Extra 60 s of etching at 139/mA/cm^2 in a solution of 1 : 1 HF (49%): EtOH lifted off the pSi films from the wafer. Ultrasonication of pSi films was then performed in an ultrasonicator water bath for 24 h to produce the pSiNPs of approximately 200 nm in diameter, which were collected via ultracentrifugation (2000 ×g for 6 min). The supernatant was also collected and then centrifuged at 20000 ×g for 10 min to retrieve the desired particles.

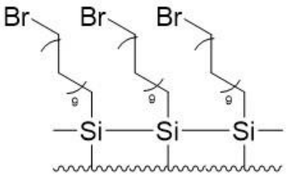

pSiNP-Br

N_2 was bubbled through 11-bromo-1-undecene (5 mL) for 20 min to remove all trace oxygen and H_2O from the system. Freshly etched pSiNPs (4 mg) was then added to the solution and the reaction mixture was allowed to be refluxed at 95°C under N_2. After 24 h, the pSiNPs were collected by centrifugation, and washed with DMF twice in order to afford **pSiNP-Br**.

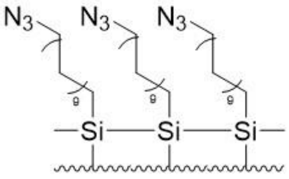

pSiNP-N_3

To the **pSiNP-Br** (4 mg) in DMF (5 mL) was added sodium azide (NaN_3, 30 mg, 10% in DMF, excess) and the resulting slurry was allowed to stir for 24 h at 60°C. After 24 h, the pSiNPs were washed with deionized H_2O three times to eliminate the excess NaN_3, affording **pSiNP-N_3**.

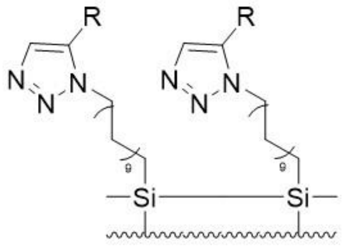

R = **PCL-DOX** or **ECL-DOX** **pSiNP-PCL-DOX** or **pSiNP-ECL-DOX**

2.4 mg of **pSiNP-N_3** was dispersed in 1.5 mL of a DMSO : H_2O (1 : 1) solution, followed by addition of a premixed solution of $CuSO_4$ (50 µL, 1 mM), THPTA (5 µL, 60 mM) and sodium ascorbate solution (25 µL, 100 mM). **PCL-DOX** (62 µL, 10 mg/mL in DMSO) or **ECL-DOX** (95 µL, 10 mg/mL in DMSO) was added to the reaction mixture and the reaction proceeded for 24 h. The pSiNPs were collected by centrifugation at 20000 ×g for 10 min and washed with DMSO (1.5 mL × 2) and H_2O (1.5 mL × 2) to eliminate the catalysts and unreacted **PCL-DOX**/**ECL-DOX**, and afford the product **pSiNP-PCL-DOX**/**pSiNP-ECL-DOX**.

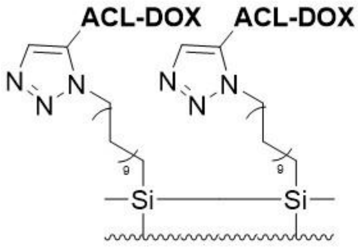

pSiNP-ACL-DOX

$CuSO_4$ (50 µL, 1 mM), THPTA (5 µL, 60 mM) and sodium ascorbate solution (50 µL, 100 mM) was mixed, the mixture and a drop of DIPEA was then added to **ACL-DOX** (49 µL, 10 mg/mL in DMSO) and **pSiNP-N_3** (2.8 mg) in 1.5 mL of a DMSO : H_2O (1 : 1) solution. After 15 min, a fresh solution of sodium ascorbate (50 µL, 100 mM) was added to the reaction mixture and the reaction proceeded for another 15 min. The pSiNPs were collected by centrifugation at 20000 ×g for 10 min and washed immediately with DMSO (1.5 mL × 2), PBS (1.5 mL × 2) and H_2O (1.5 mL × 2) to afford the product **pSiNP-ACL-DOX**.

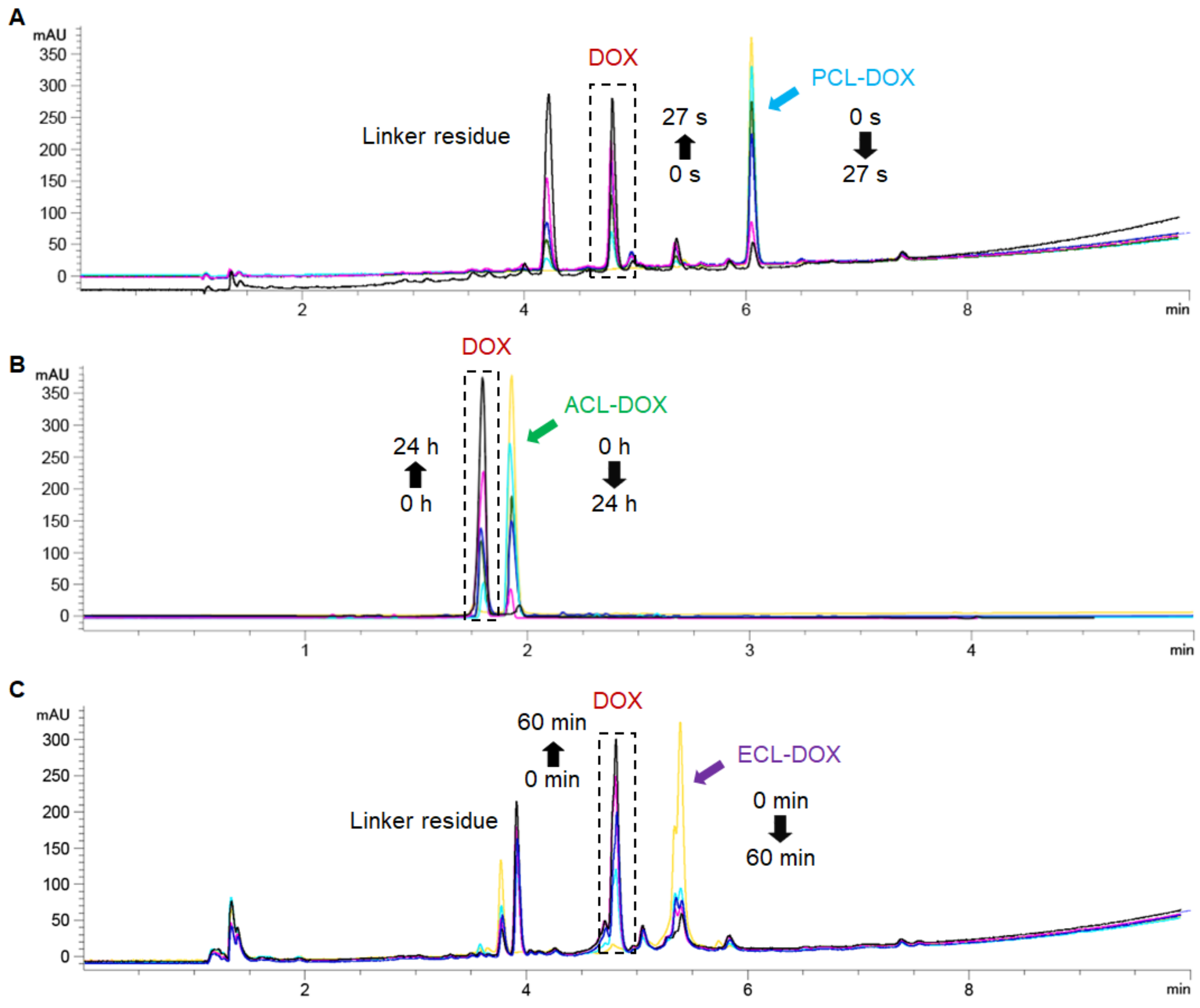

Figure S1. (A) HPLC chromatograms of **PCL-DOX** upon exposure to photo-irradiation (λ = 400 nm, 849 mW/cm^2) in PBS at 37°C for 0, 3, 9, 15, 21 and 27 s. (B) HPLC chromatograms of **ACL-DOX** upon exposure to sodium acetate buffer at 37°C (pH = 5.2) for 0, 1, 3, 5, 10 and 24 h. (C) HPLC chromatograms of **ECL-DOX** upon exposure to β-glucuronidase (250 U/mL) in PBS at 37°C for 0, 10, 30, 60 and 90 min.

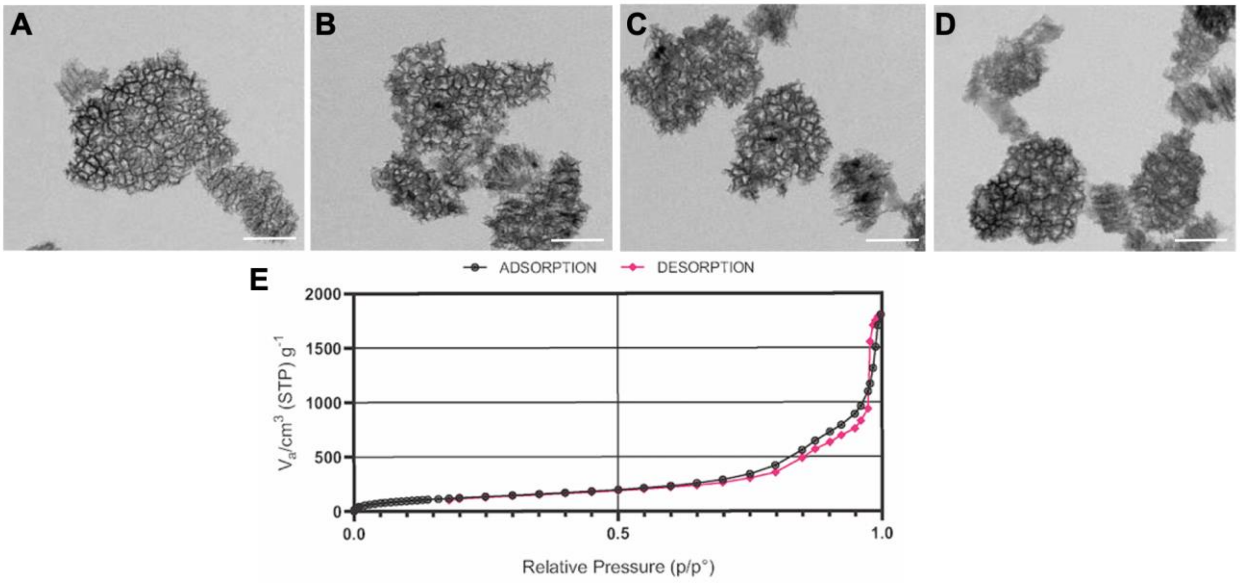

Figure S2. (A) TEM images of **pSiNP-N_3**; (B) TEM images of **pSiNP-PCL-DOX**; (C) TEM images of **pSiNP-ACL-DOX**; (D) TEM images of **pSiNP-ECL-DOX**. (E) BET surface area and pore size measured by BET. TEM image Scale bar = 100 nm.

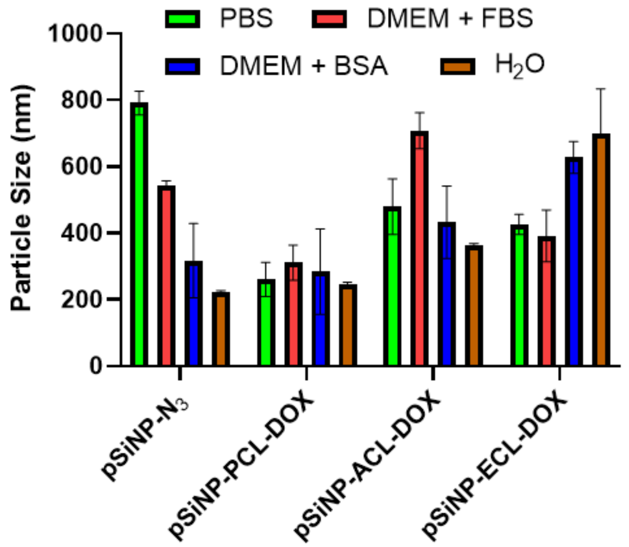

Figure S3. Particle size measured by DLS of **pSiNP-N$_3$**, **pSiNP-PCL-DOX**, **pSiNP-ACL-DOX** and **pSiNP-ECL-DOX** in PBS, DMEM + FBS and DMEM + BSA. Data shown as mean ± S.D. (N = 3).

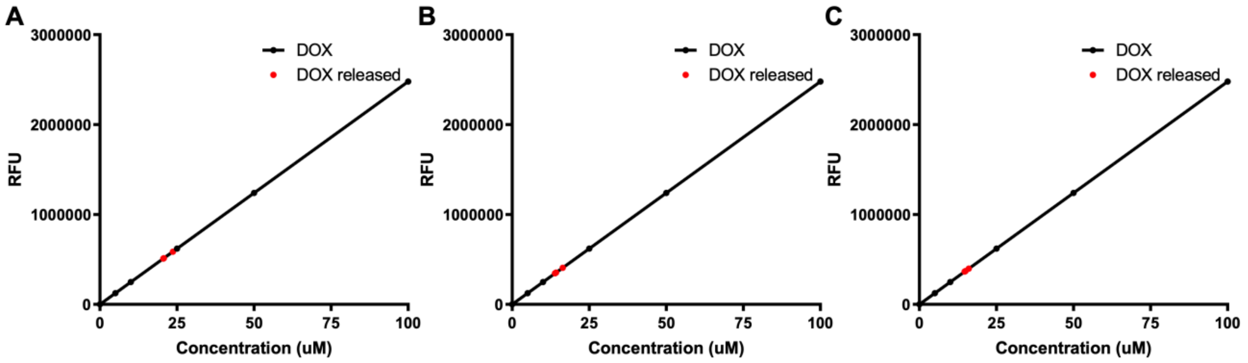

Figure S4. Fluorescence intensity of supernatant (100 µL, 60 µg/mL) collected from (A) **pSiNP-PCL-DOX**, (B) **pSiNP-ACL-DOX** and (C) **pSiNP-ECL-DOX** after exposure to the corresponding stimulus and treated with thorough sonication wash. Data are shown as mean ± S.D (N = 3).

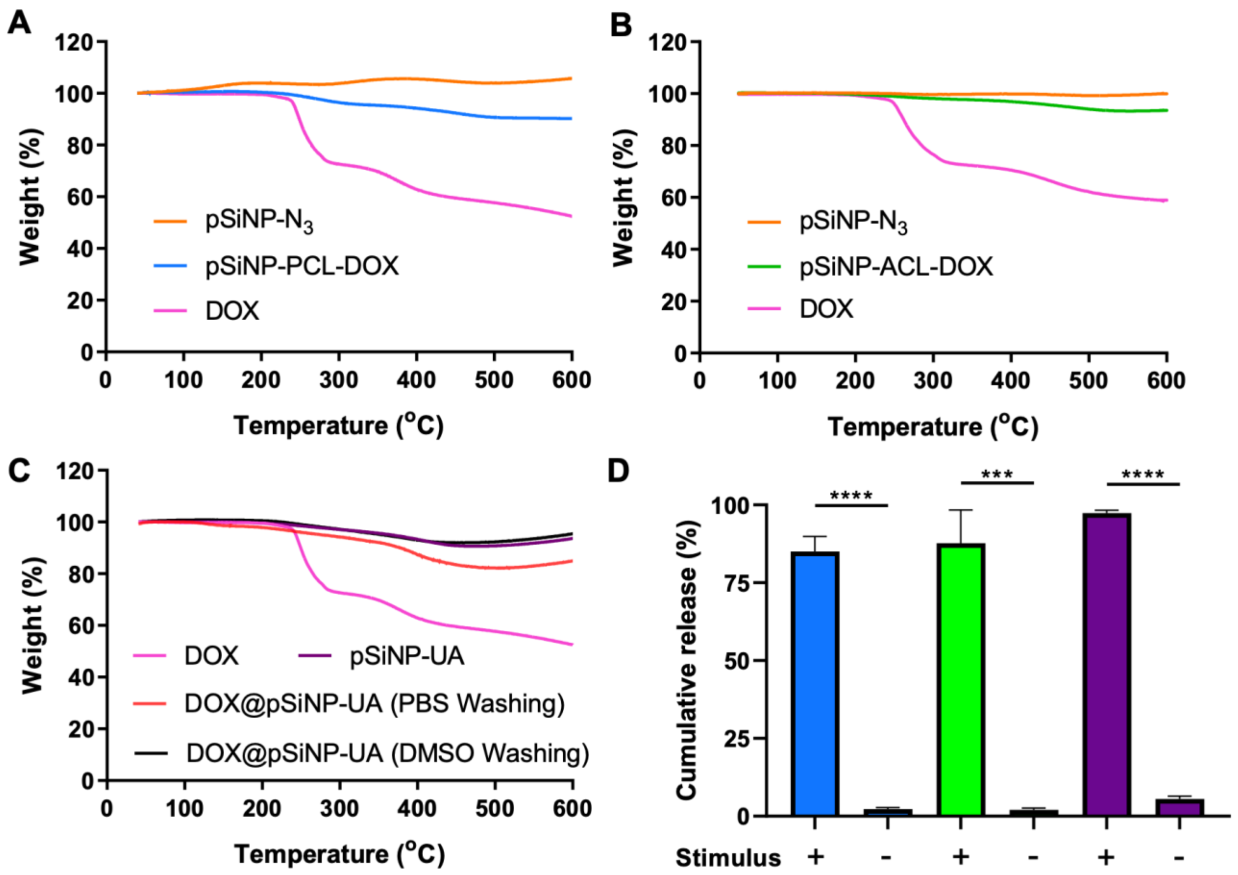

Figure S5. Loading capacity and controlled release analysis of the pSiNPs. Thermogravimetric analysis (TGA) of (A) **pSiNP-PCL-DOX**, **pSiNP-N_3** and DOX. (B) TGA of **pSiNP-ACL-DOX**, **pSiNP-N_3** and DOX. (C) TGA of **DOX@pSiNP-UA** (undecylenic acid-functionalized pSiNPs with DOX adsorptively loaded and then washed with either PBS/DMSO), **pSiNP-UA** and DOX. (D) Cumulative release profile of **pSiNP-PCL-DOX** (PBS), **pSiNP-ACL-DOX** (PBS or sodium acetate buffer) and **pSiNP-ECL-DOX** (PBS) in the presence (+) or absence (-) of corresponding stimulus after 48 h at 37°C. Data shown as mean ± S.D. (N = 3). Student's *t*-test, *P**<0.1, *P***<0.01, *P****<0.001, *P*****<0.0001.

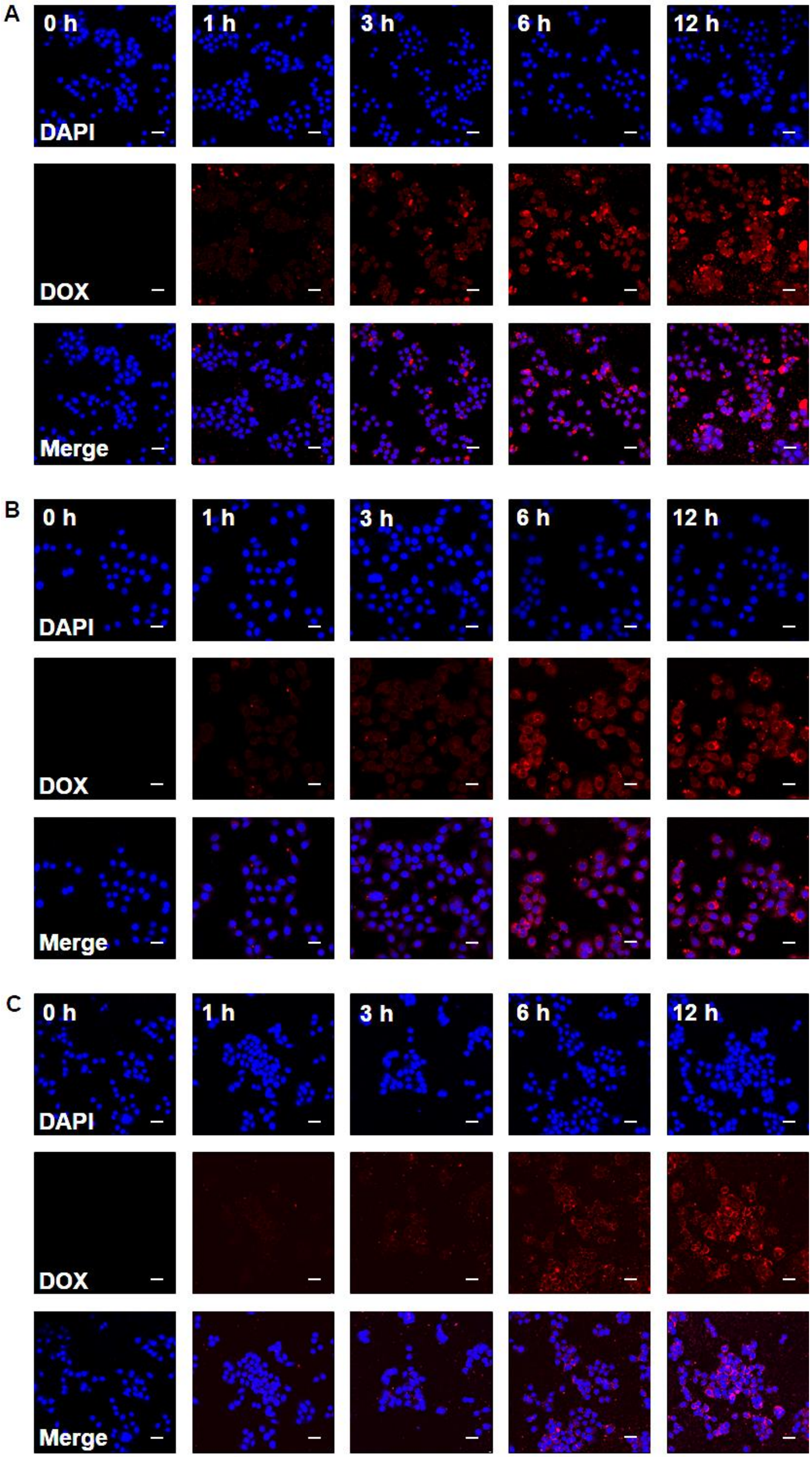
A
0 h
1 h
3 h
6 h
12 h
DAPI
DOX
Merge
B
0 h
1 h
3 h
6 h
12 h
DAPI
DOX
Merge
C
0 h
1 h
3 h
6 h
12 h
DAPI
DOX
Merge

Figure S6. The confocal microscopy studies of the cellular uptake and controlled release behaviors of (A) **pSiNP-PCL-DOX**, (B) **pSiNP-ACL-DOX** and (C) **pSiNP-ECL-DOX** after incubating with HeLa cells for 0 h, 1 h, 3 h, 6 h and 12 h in the presence of corresponding stimulus at 37°C. DAPI (blue fluorescence) was used to stain the cell nucleus. Scale bar = 20 μm.

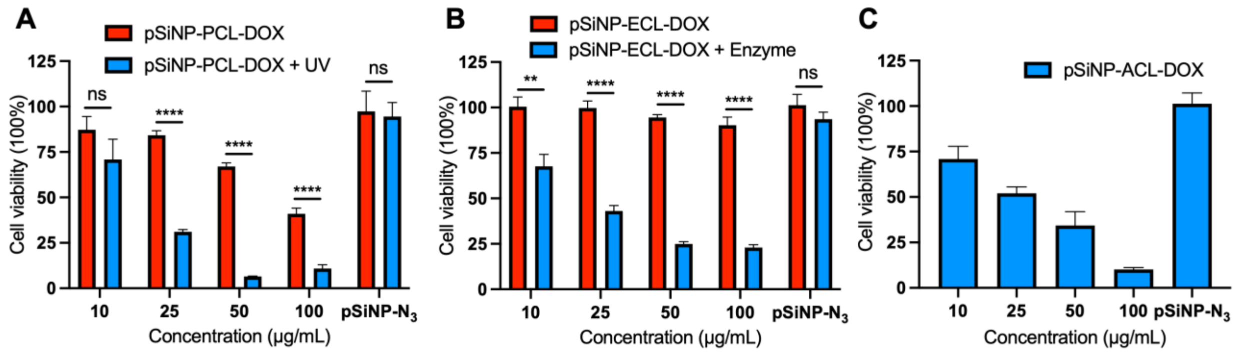

Figure S7. Cell viability assay of C32 upon the treatment of three pSiNP-SCL-DOXs. (A) Cell viability of C32 cells treated with different concentrations of **pSiNP-PCL-DOX** for 48 h, with (red) or without (blue) photo-irradiation. (B) Cell viability of C32 cells treated with different concentrations of **pSiNP-ECL-DOX** for 48 h, with (red) or without (blue) β-glucuronidase incubation. (C) Cell viability of C32 cells treated with different concentrations of **pSiNP-ACL-DOX** 48 h at physiological pH. Data shown as mean ± S.D. (N = 4). Student's *t*-test, ns: not significant, *P***<0.01, *P****<0.001, *P*****<0.0001.

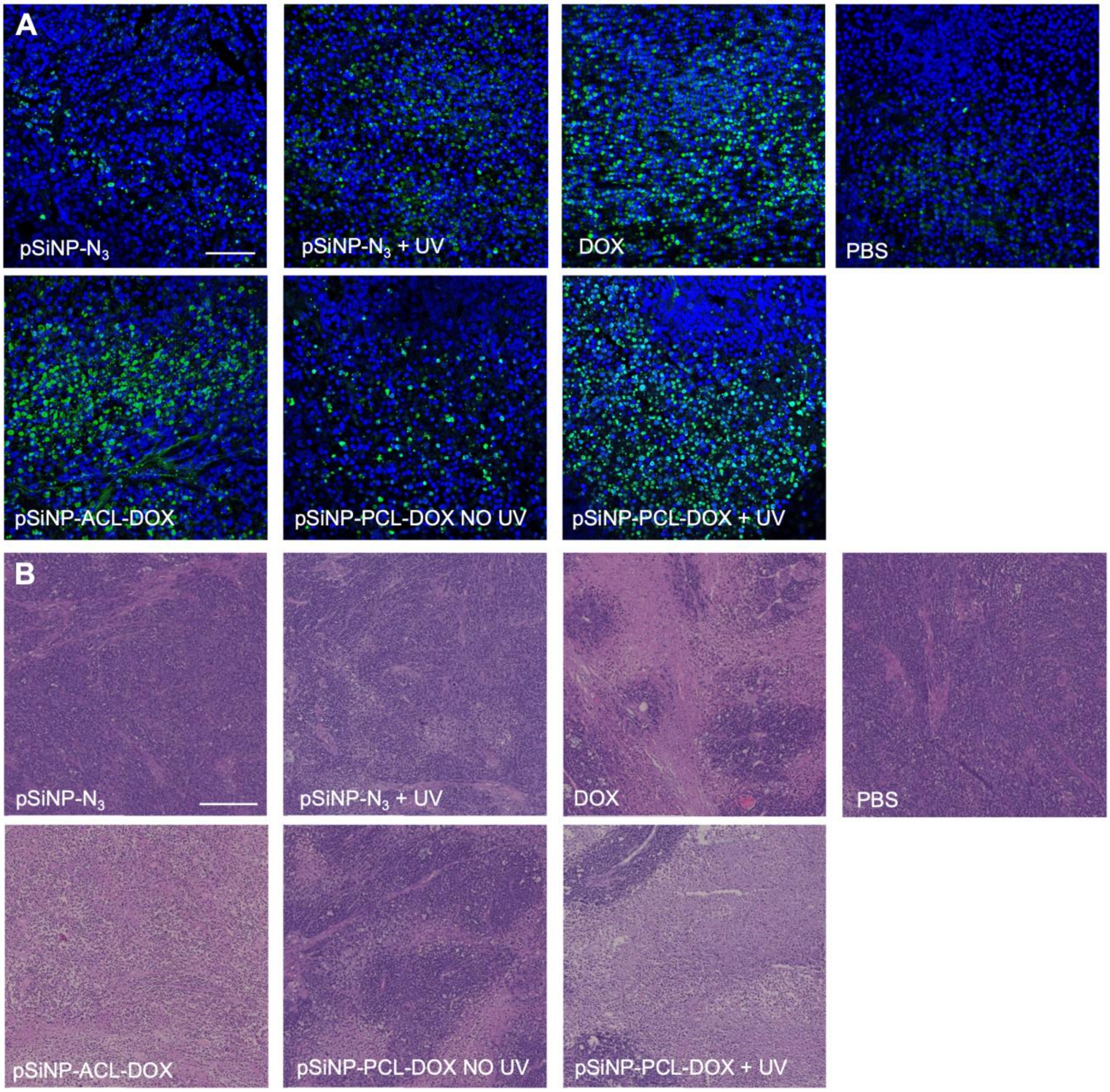

Figure S8. (A) TUNEL and (B) H&E staining of HeLa tumor tissues from the mice treated with **pSiNP-N_3** (no photo-irradiation), **pSiNP-N_3** + photo-irradiation, DOX, **pSiNP-ACL-DOX**, **pSiNP-PCL-DOX** (no photo-irradiation), **pSiNP-PCL-DOX** + photo-irradiation and PBS on day 24, depicting apoptosis (TUNEL) and cell death (H&E). In (A) the green spots represent TUNEL-positive cells (apoptosis) and the blue spots represent cell nuclei. Scale bar = 250 µm.

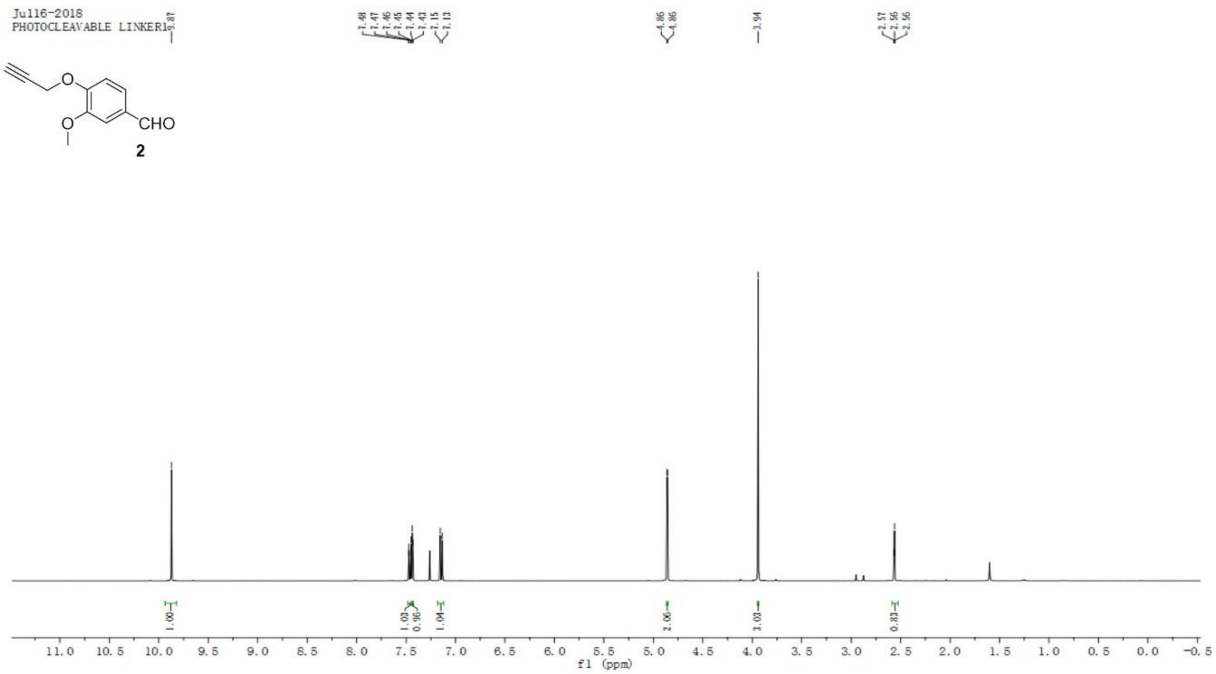

Figure S9. ^{1}H NMR spectrum of compound **2**.

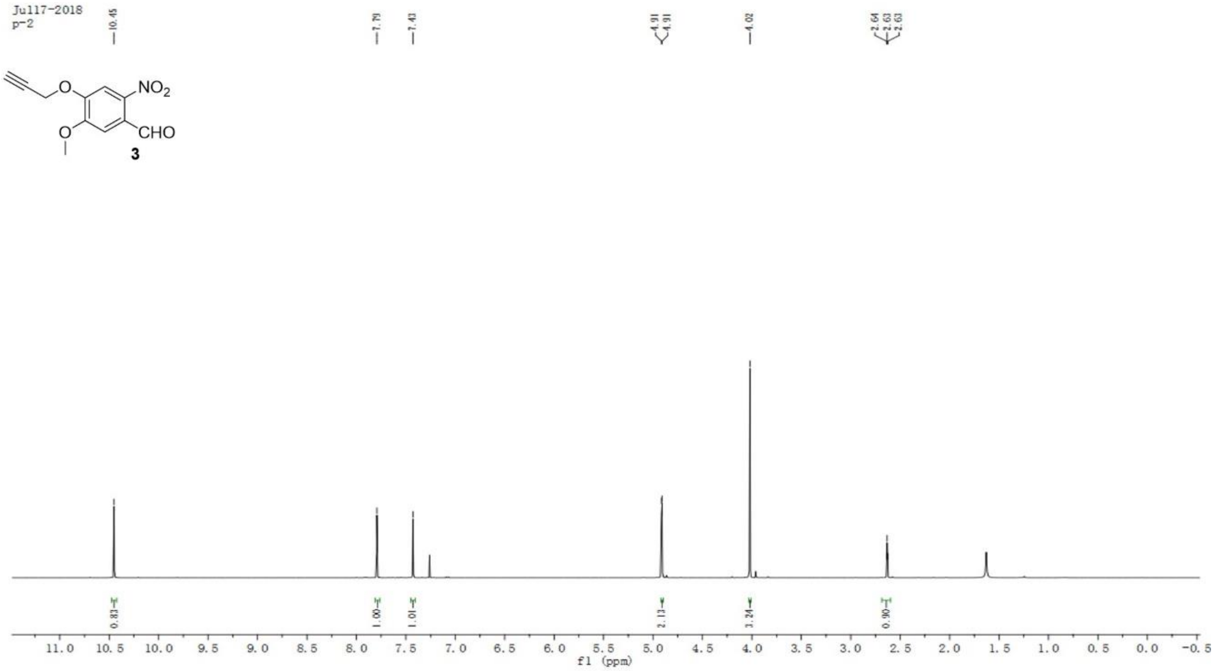

Figure S10. ^{1}H NMR spectrum of compound **3**.

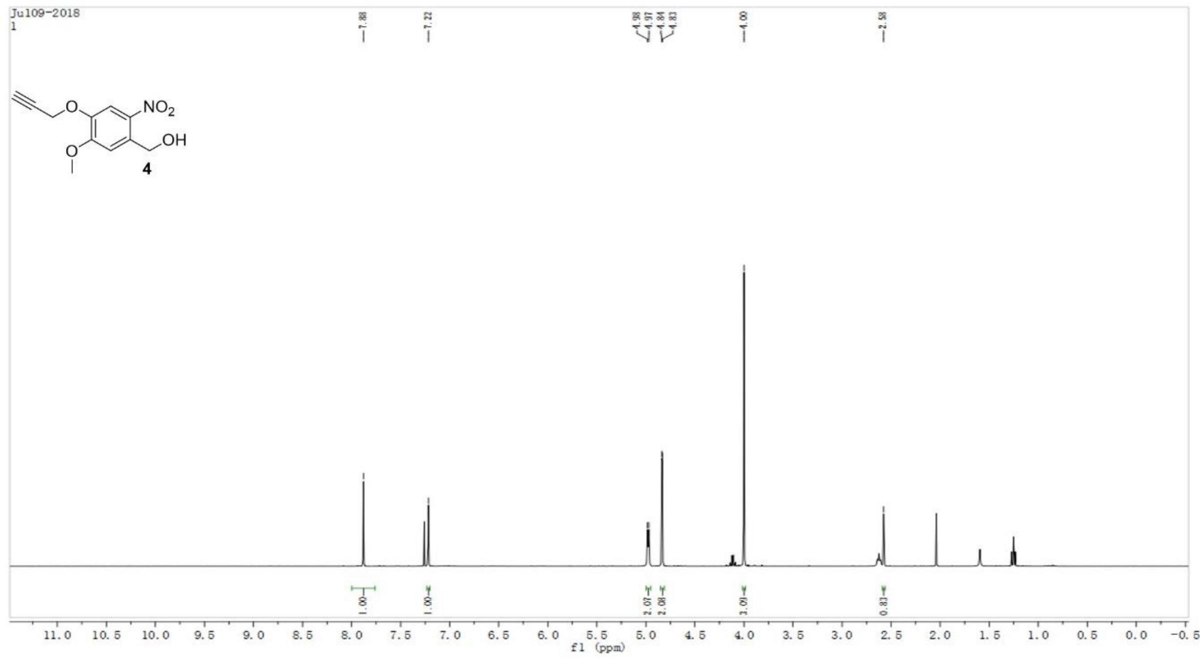

Figure S11. ^{1}H NMR spectrum of compound **4**.

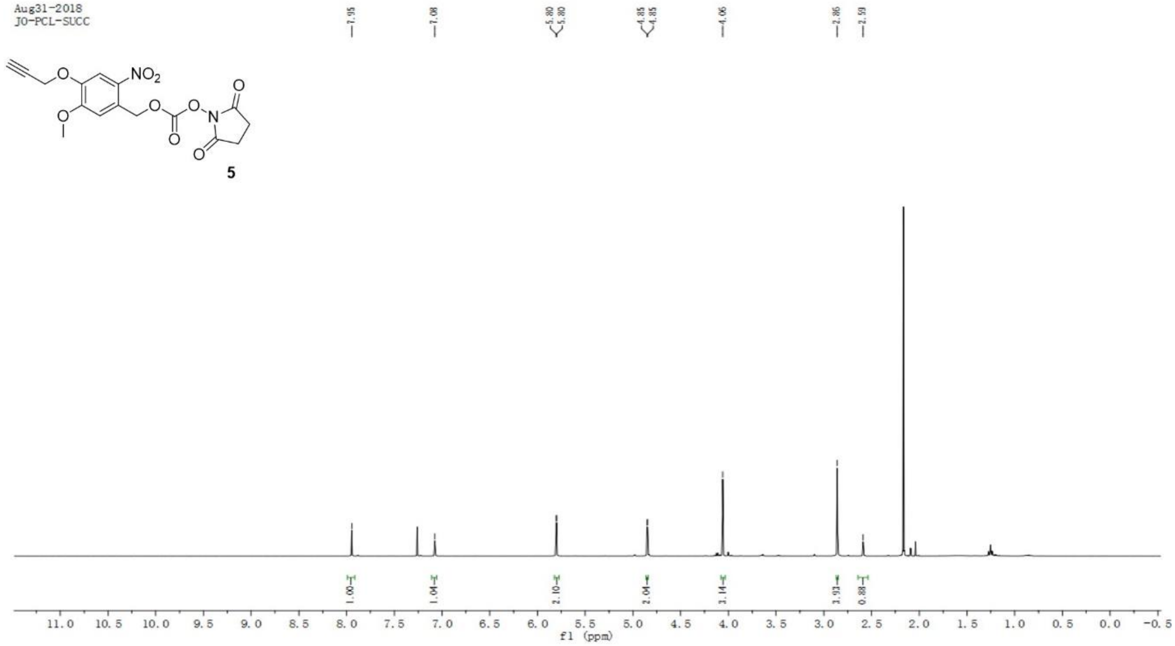

Figure S12. ^{1}H NMR spectrum of compound **5**.

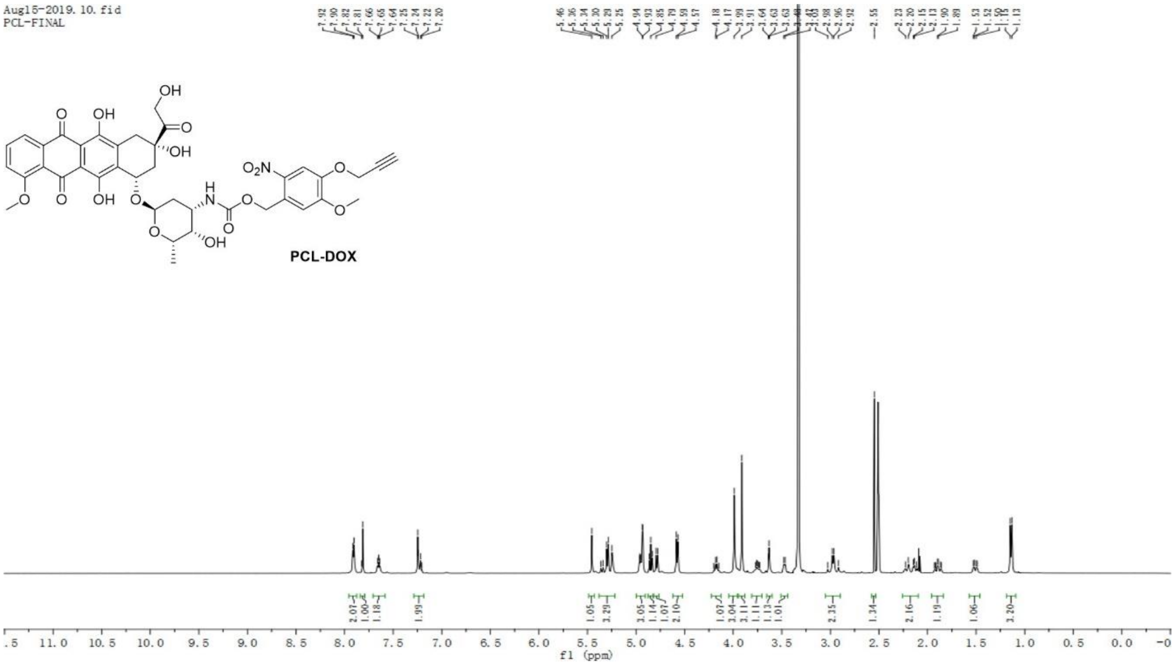

Figure S13. ^{1}H NMR spectrum of **PCL-DOX**.

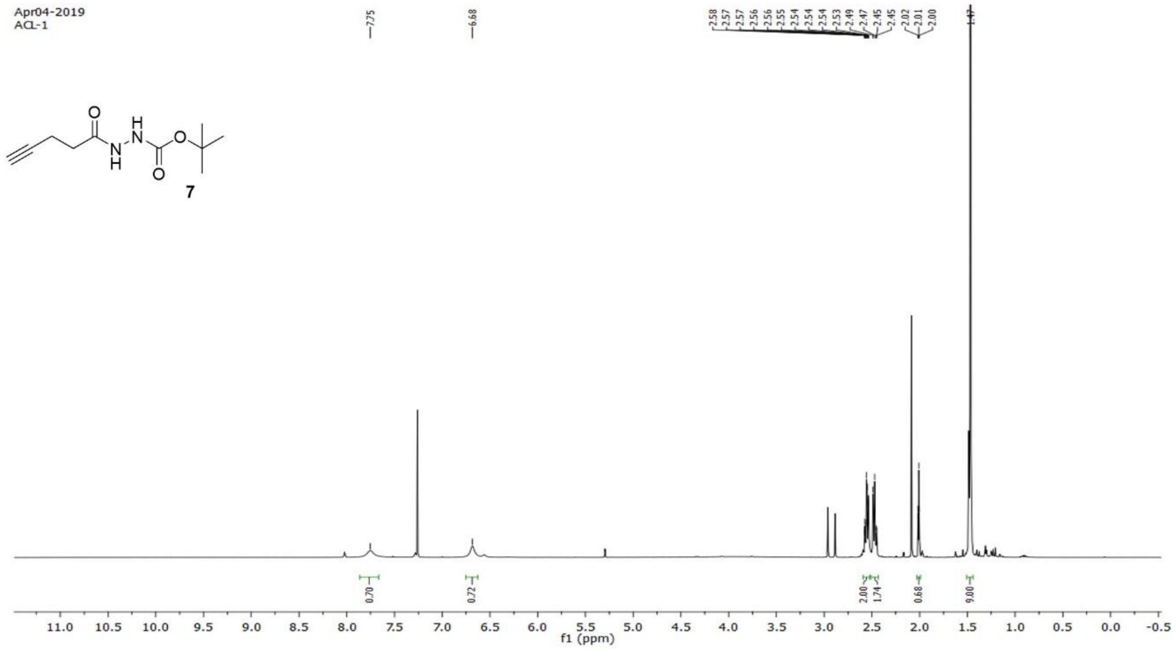

Figure S14. ^{1}H NMR spectrum of compound **7.**

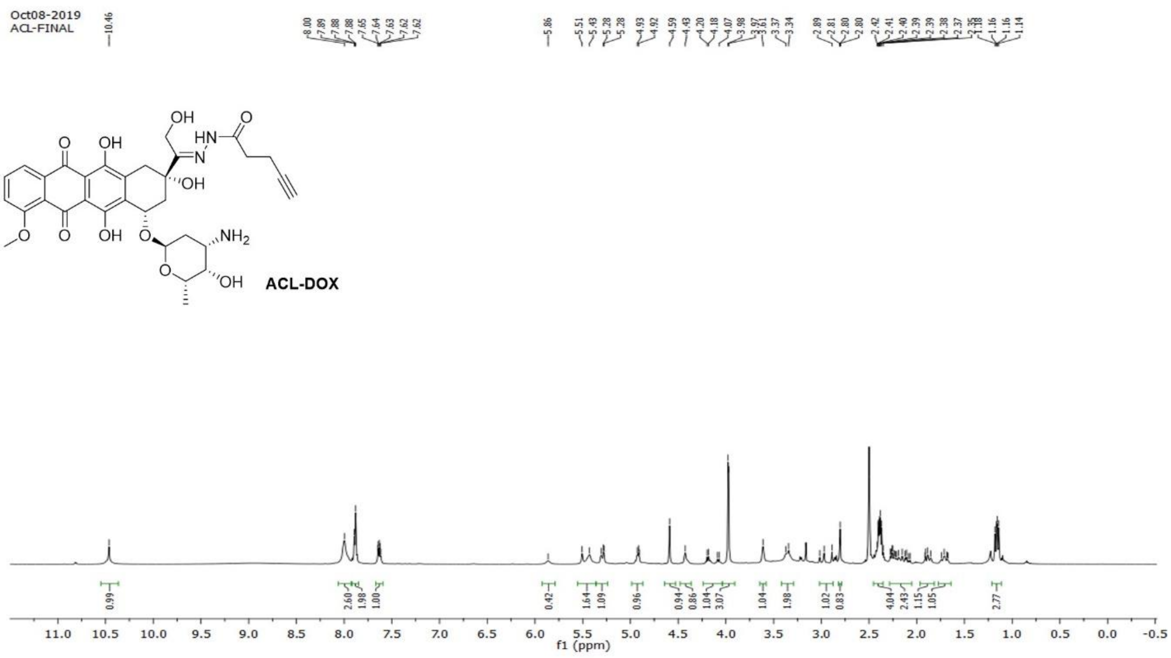

Figure S15. ^{1}H NMR spectrum of **ACL-DOX**.

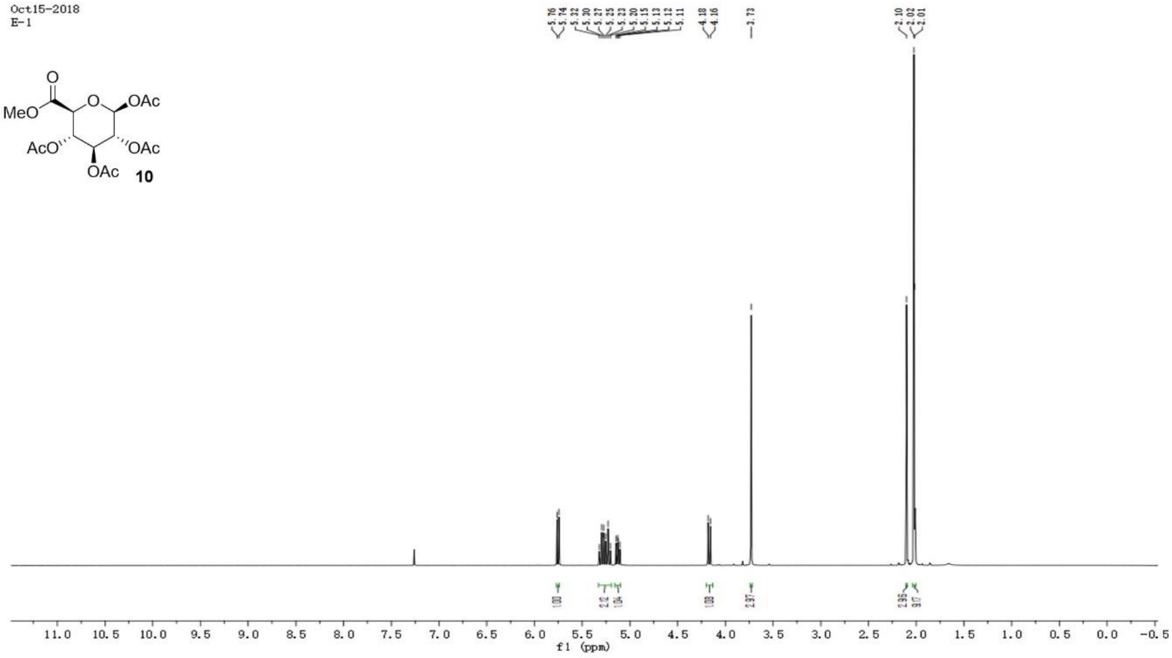

Figure S16. ^{1}H NMR spectrum of compound **10.**

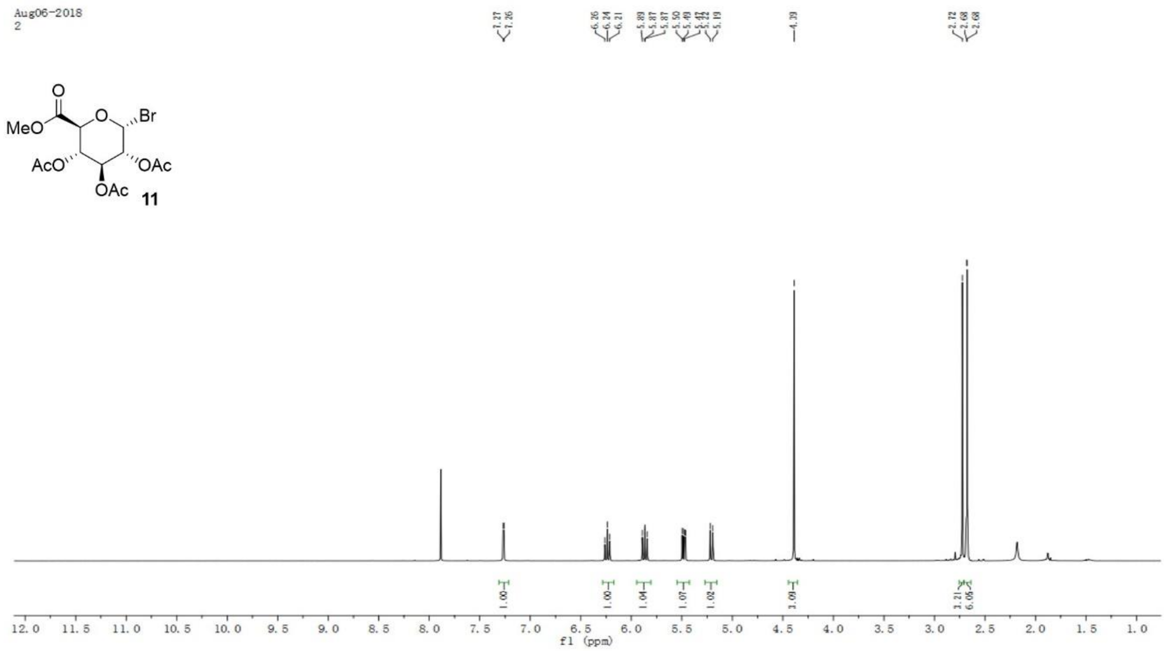

Figure S17. ^{1}H NMR spectrum of compound **11**.

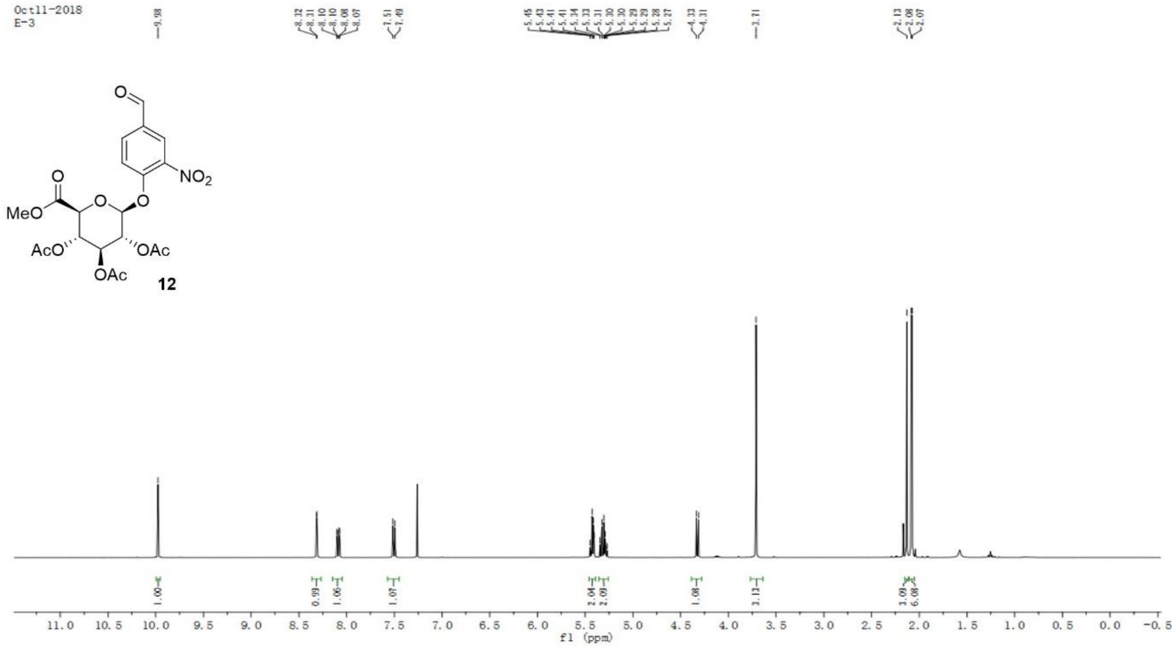

Figure S18. ^{1}H NMR spectrum of compound **12.**

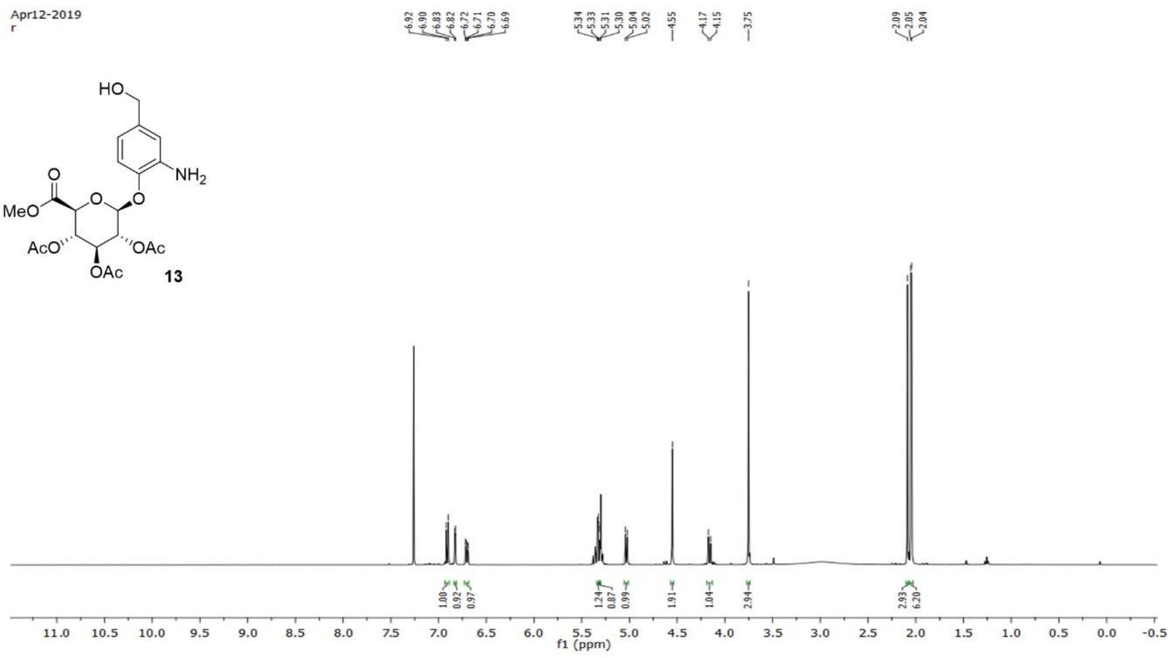

Figure S19. ^{1}H NMR spectrum of compound **13**.

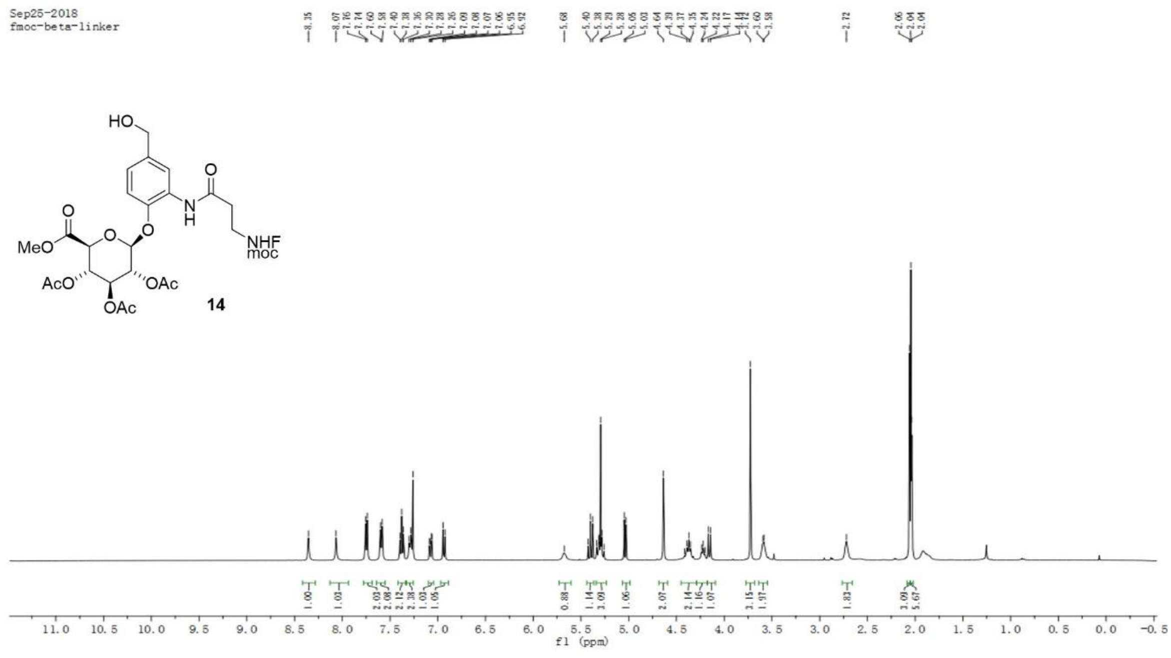

Figure S20. ^{1}H NMR spectrum of compound **14.**

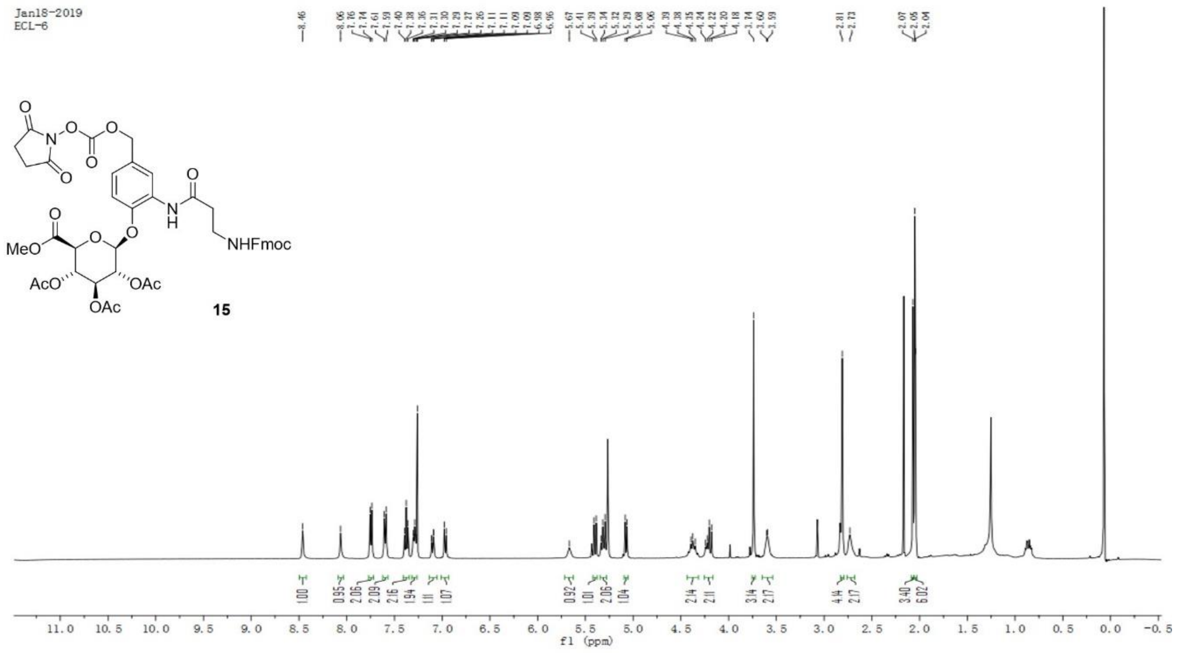

Figure S21. ^{1}H NMR spectrum of compound **15**.

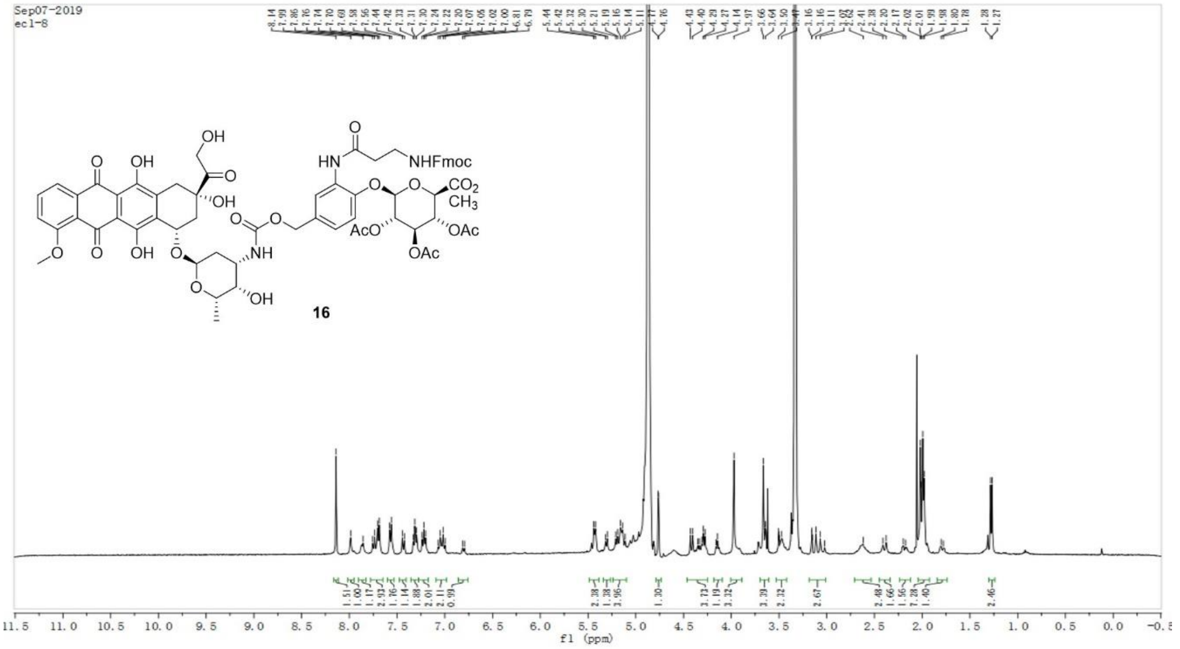

Figure S22. ^{1}H NMR spectrum of compound **16.**

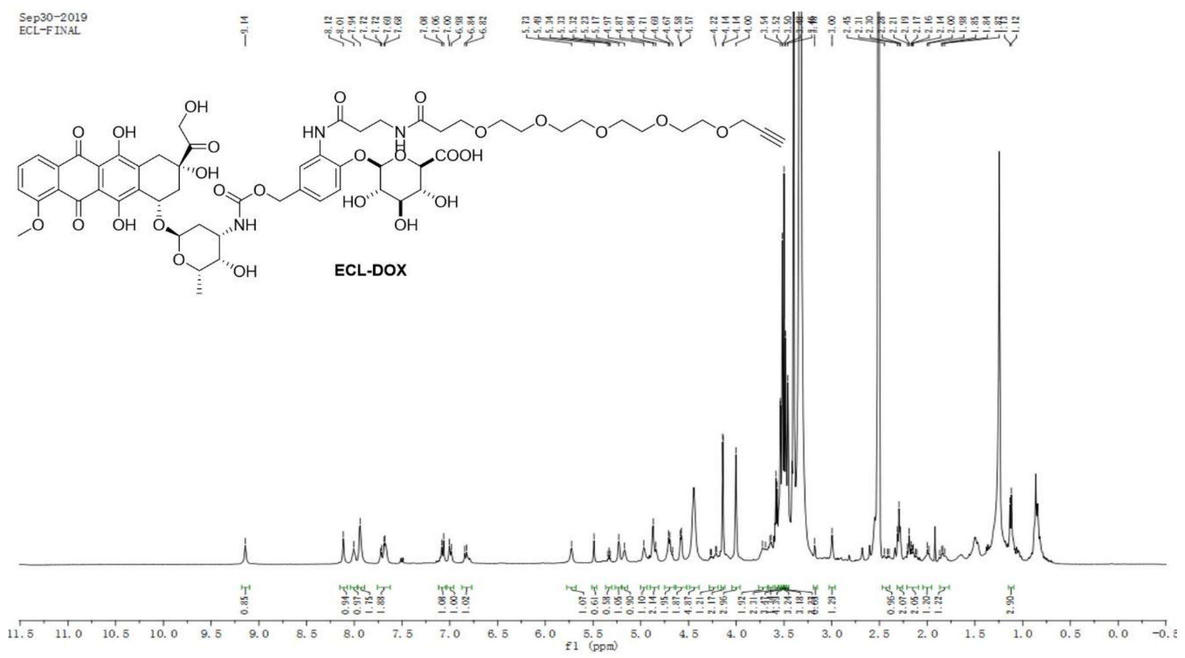

Figure S23. ^{1}H NMR spectrum of **ECL-DOX.**

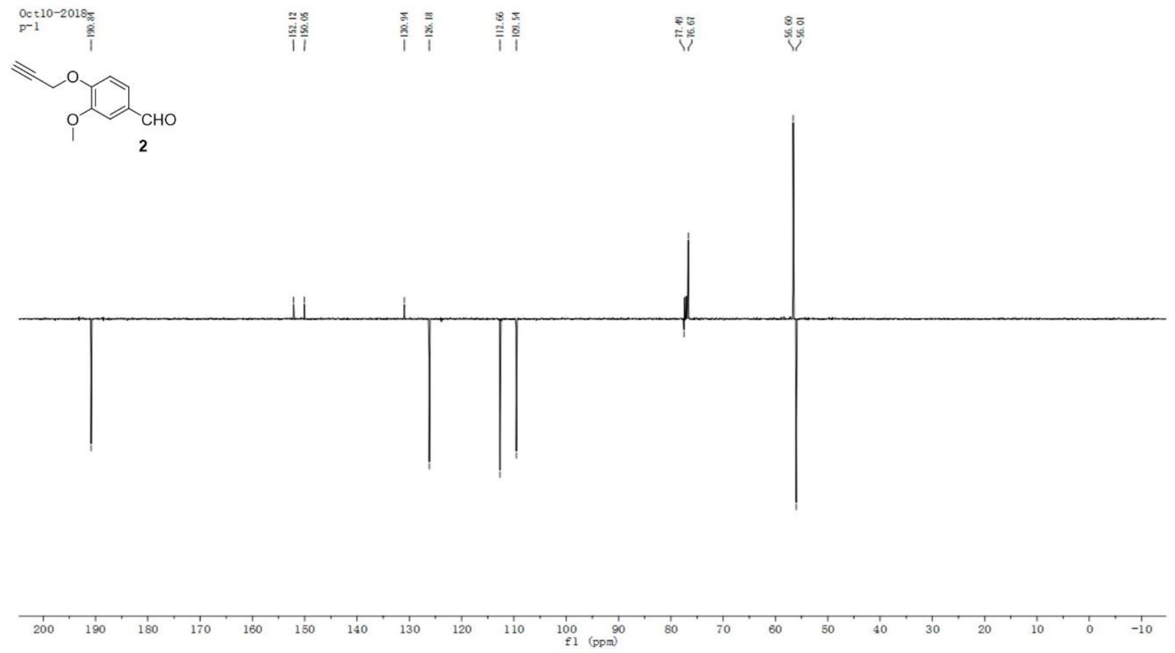

Figure S24. ^{13}C (DEPT) NMR spectrum of compound **2.**

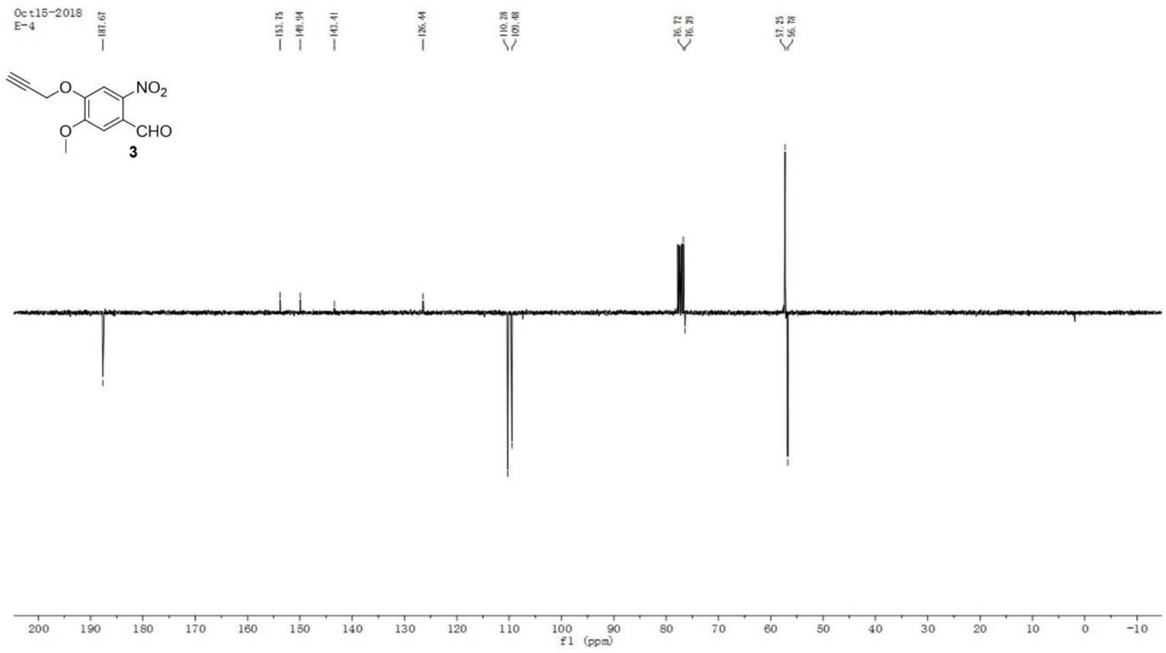

Figure S25. ^{13}C (DEPT) NMR spectrum of compound **3.**

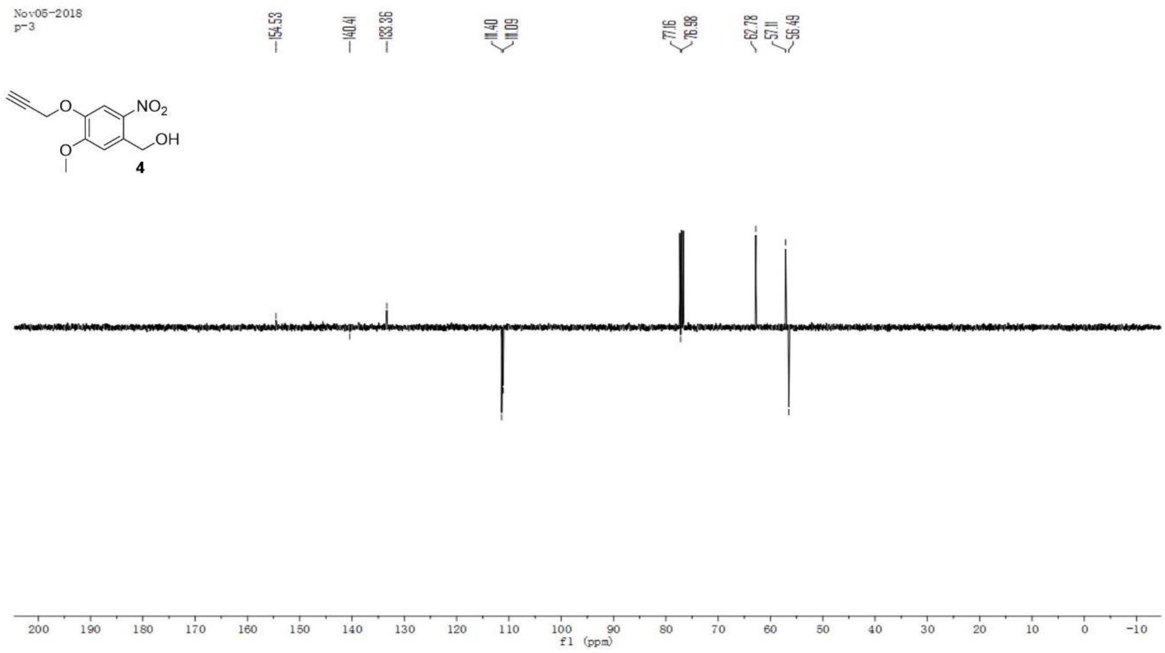

Figure S26. ^{13}C (DEPT) NMR spectrum of compound **4.**

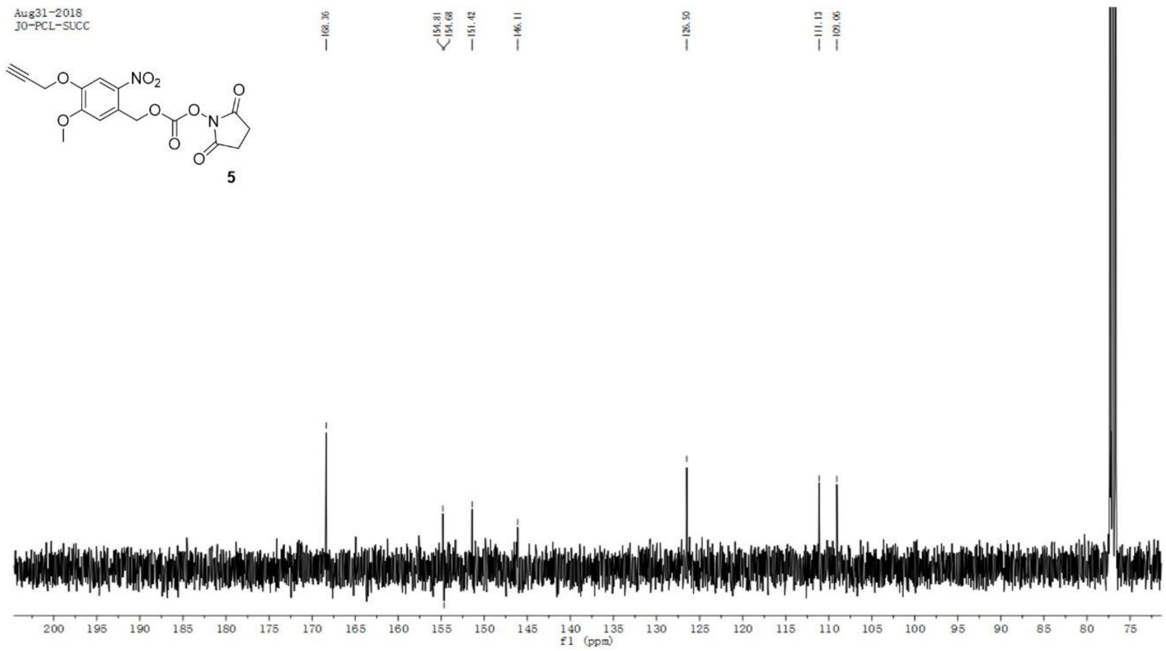

Figure S27. ^{13}C NMR spectrum of compound **5**.

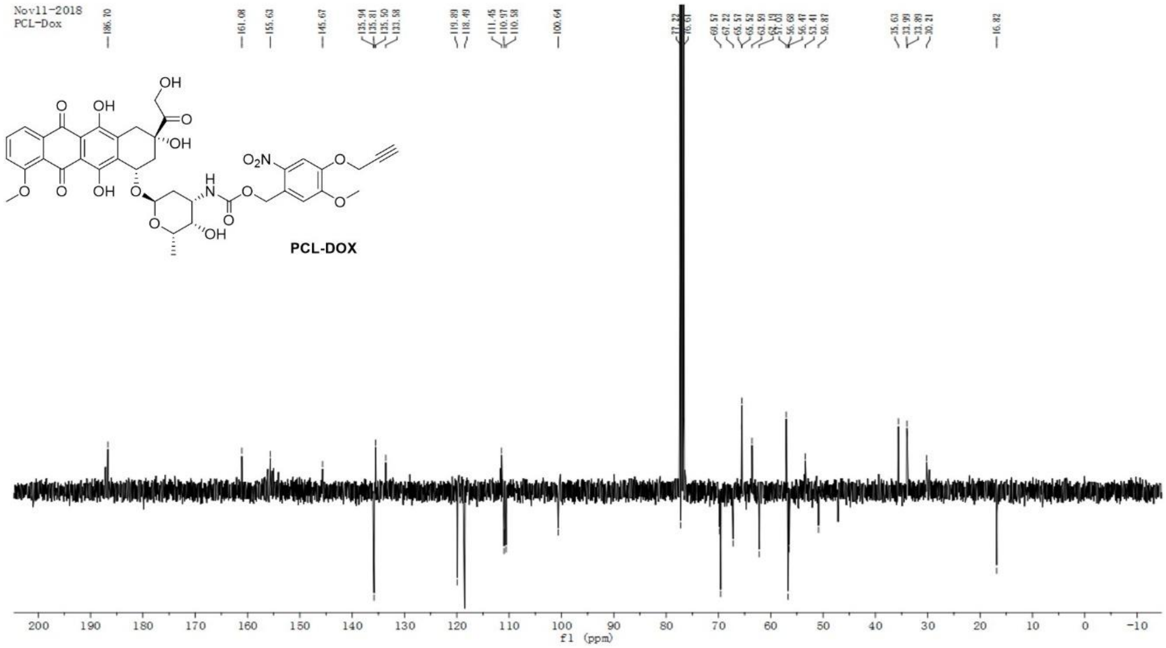

Figure S28. ^{13}C (DEPT) NMR spectrum of **PCL-DOX.**

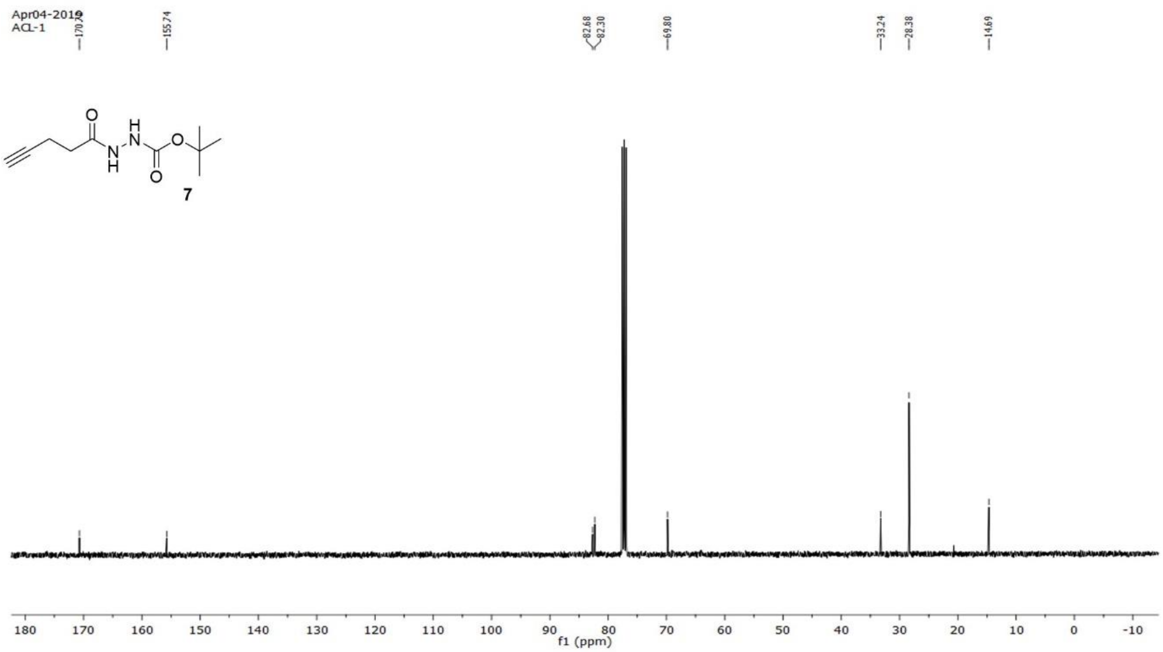

Figure S29. ^{13}C NMR spectrum of compound **7.**

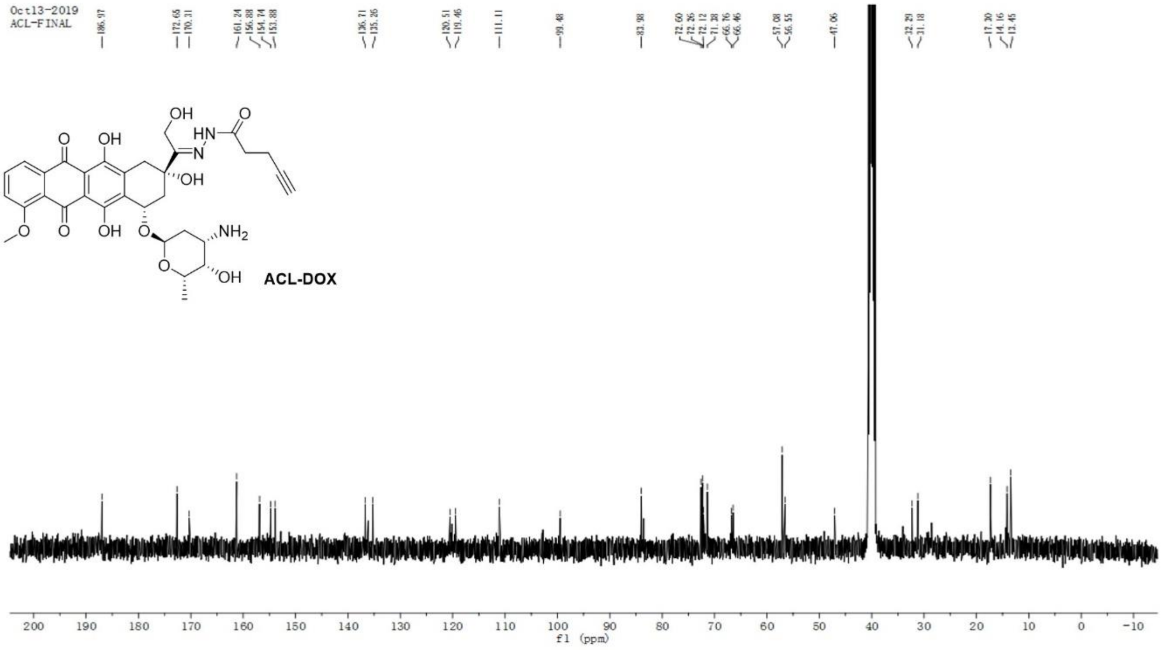

Figure S30. ^{13}C NMR spectrum of **ACL-DOX.**

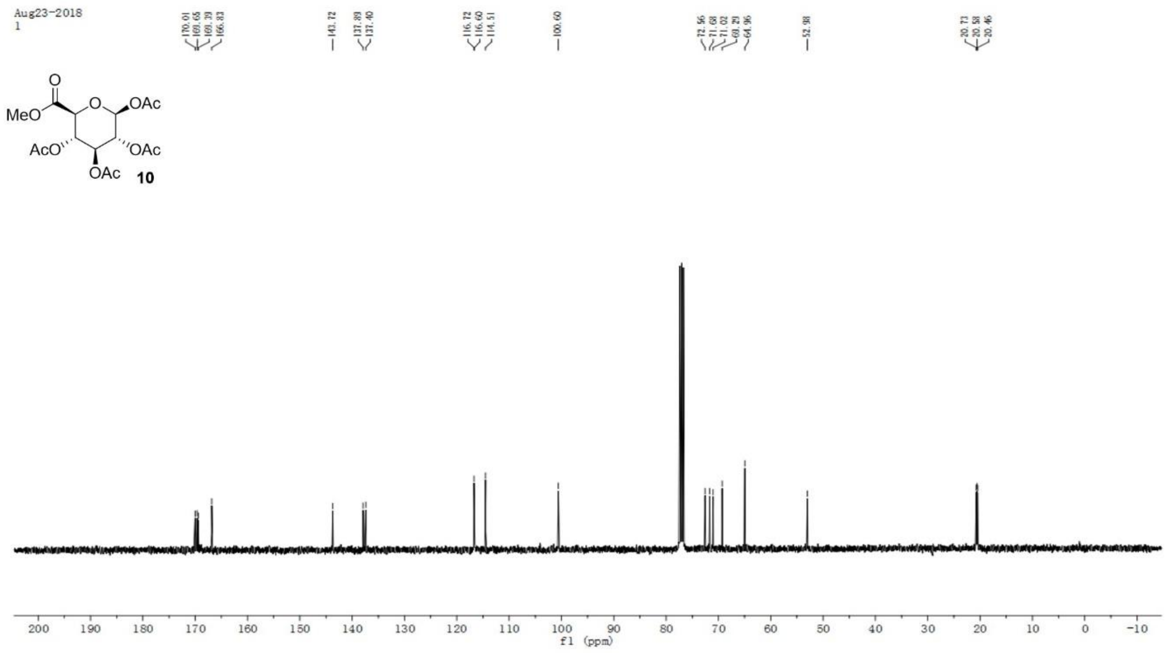

Figure S31. ^{13}C NMR spectrum of compound **10**.

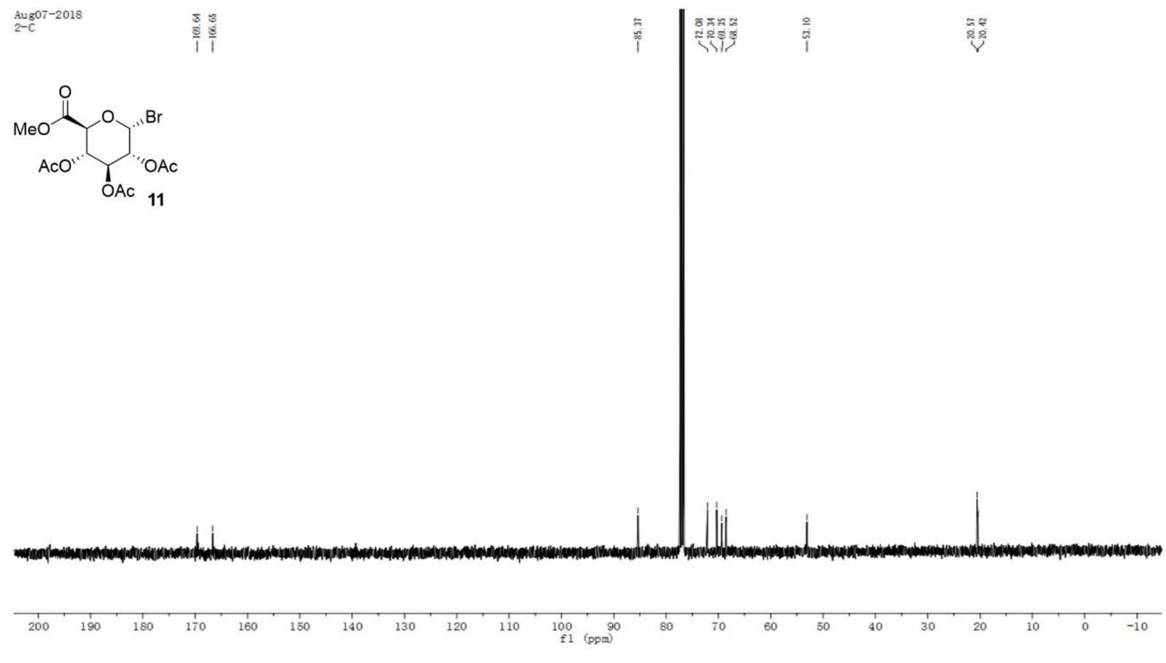

Figure S32. ^{13}C NMR spectrum of compound **11.**

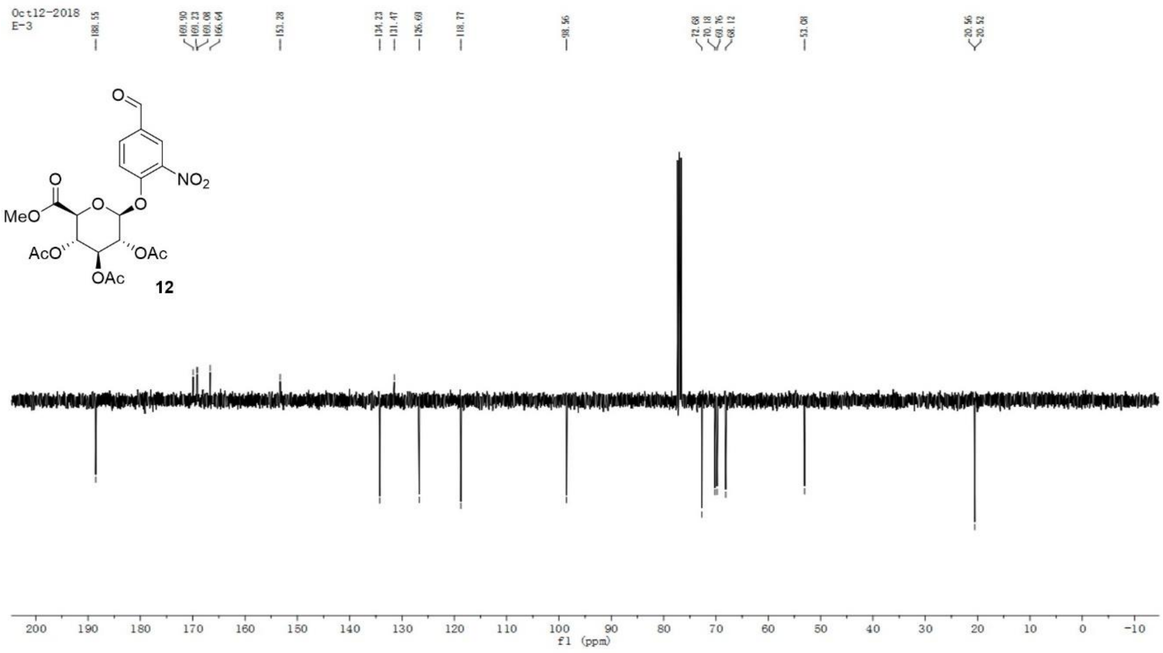

Figure S33. ^{13}C NMR spectrum of compound **12**.

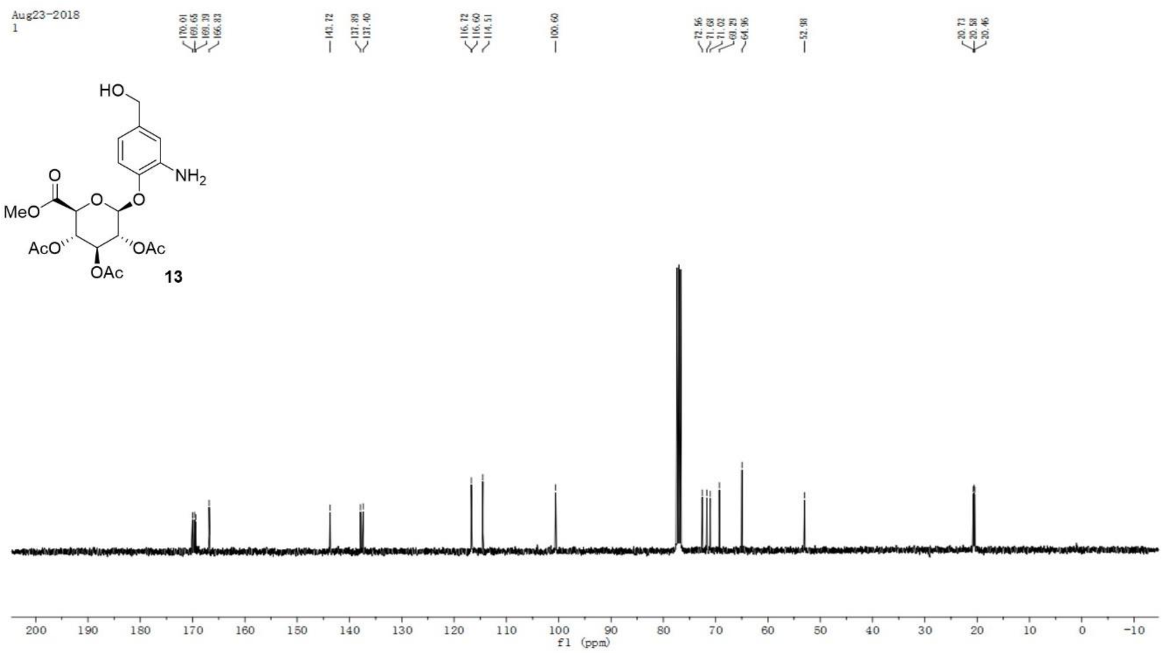

Figure S34. ^{13}C NMR spectrum of compound **13**.

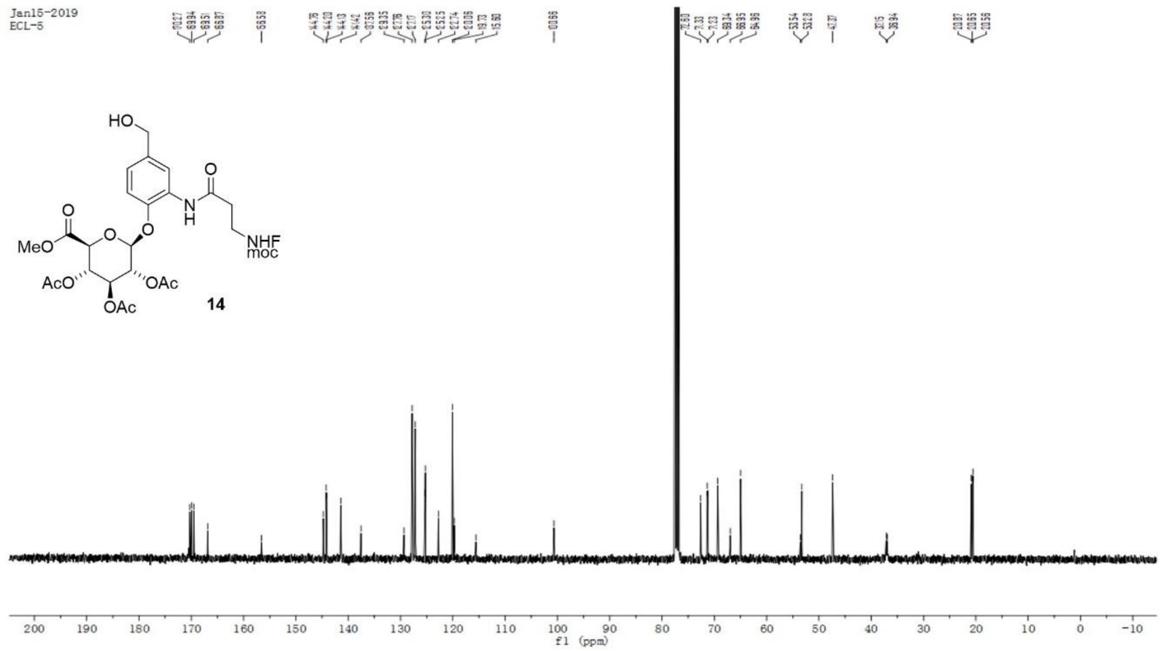

Figure S35. ^{13}C NMR spectrum of compound **14**.

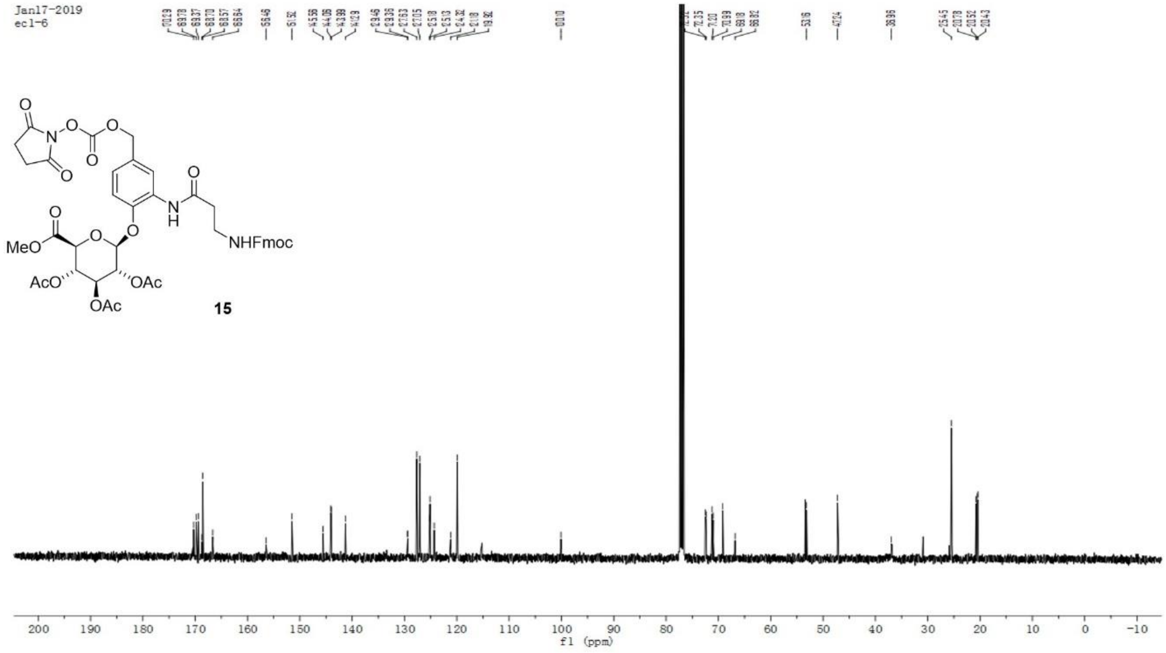

Figure S36. ^{13}C NMR spectrum compound **15**.

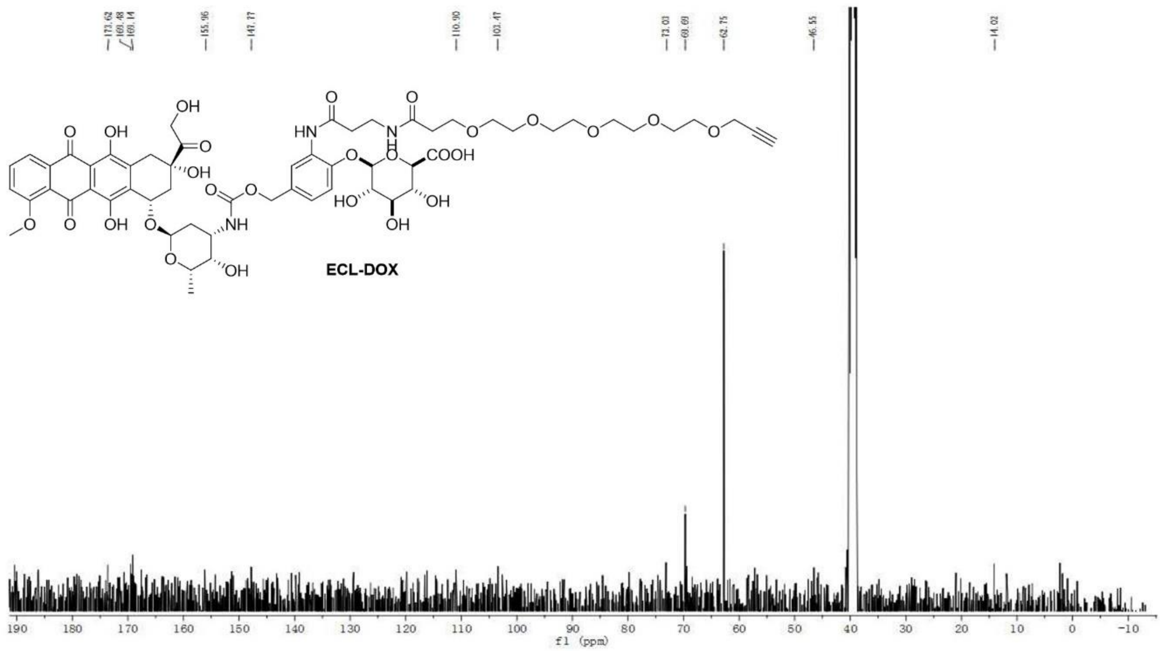

Figure S37. ^{13}C NMR spectrum of **ECL-DOX**.

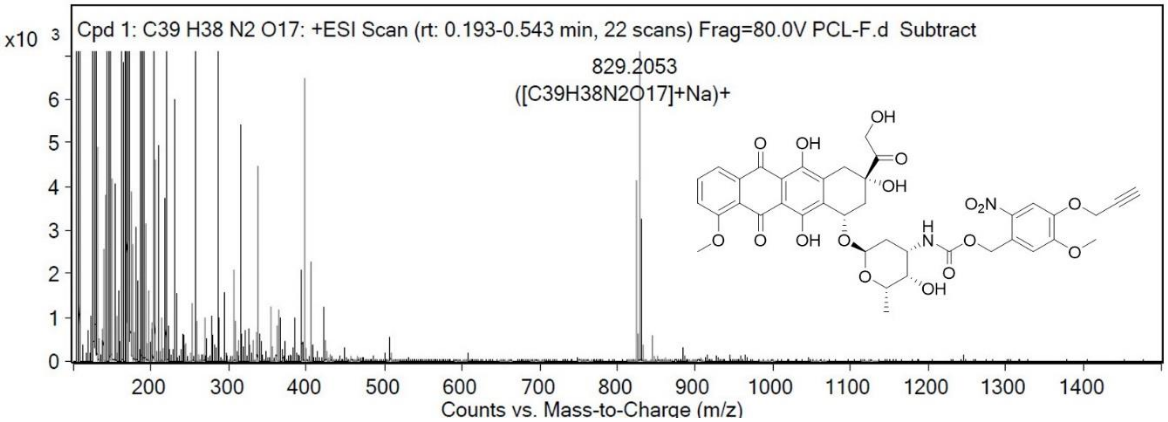

Figure S38. High resolution mass spectrum (HRMS) spectra of **PCL-DOX**.

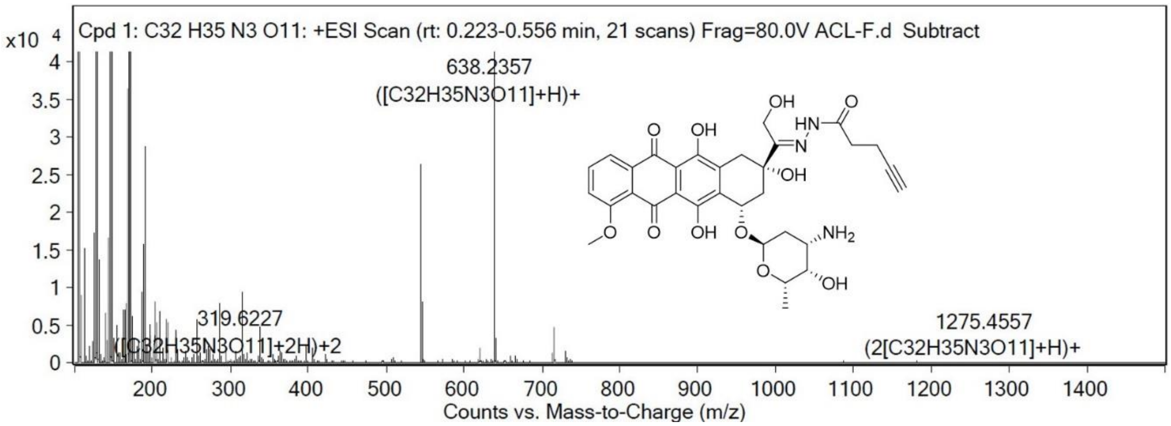

Figure S39. High resolution mass spectrum (HRMS) spectra of **ACL-DOX**.

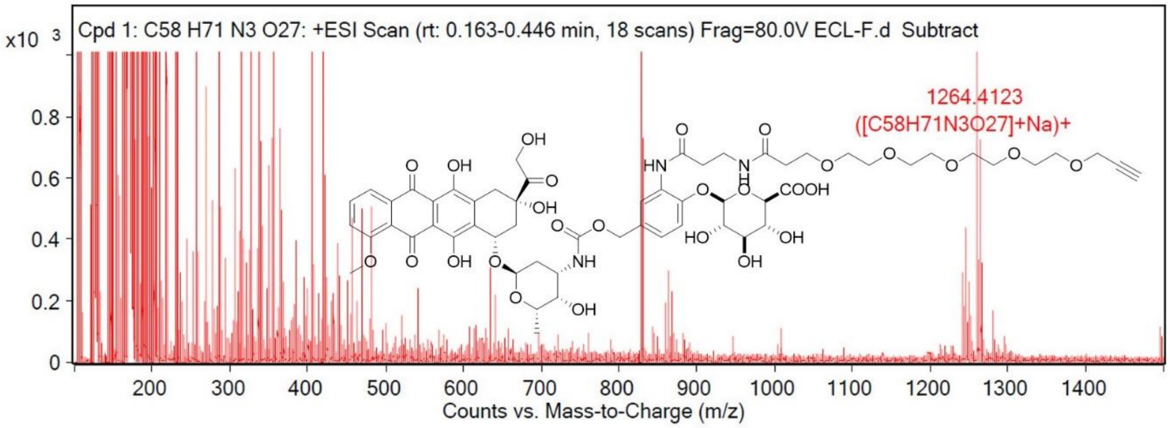

Figure S40. High resolution mass spectrum (HRMS) spectra of **ECL-DOX.**

[1] a) S. K. Choi, M. Verma, J. Silpe, R. E. Moody, K. Tang, J. J. Hanson, J. R. Baker, Jr., *Bioorg. Med. Chem.*, **2012**, *20*, 1281-1290; b) J. D. Wallat, J. K. Harrison, J. K. Pokorski, *Mol. Pharm.*, **2018**, *15*, 2954-2962; c) P. J. Burke, J. Z. Hamilton, T. A. Pires, J. R. Setter, J. H. Hunter, J. H. Cochran, A. B. Waight, K. A. Gordon, B. E. Toki, K. K. Emmerton, W. Zeng, I. J. Stone, P. D. Senter, R. P. Lyon, S. C. Jeffrey, *Mol. Cancer Ther.*, **2016**, *15*, 938-945.

[2] C. G. Bavnhoj, M. M. Knopp, C. M. Madsen, K. Lobmann, *Int. J. Pharm. X*, **2019**, *1*, 100008.

[3] T. Tieu, S. Dhawan, V. Haridas, L. M. Butler, H. Thissen, A. Cifuentes-Rius, N. H. Voelcker, *ACS Appl. Mater. Interfaces*, **2019**, *11*, 22993-23005.

[4] M. Ruike, M. Houzouji, A. Motohashi, N. Murase, A. Kinoshita, K. Kaneko, *Langmuir*, **1996**, *12*, 4828-4831.
